# Dinuclear Copper(II) Complexes of 2,6-Bis[(*N*-Methylpiperazine-1-yl)methyl]-4-Formyl Phenol Ligand: Promising Biomimetic Catalysts for Dye Residue Degradation and Drug Synthesis

**DOI:** 10.3390/ijms26041603

**Published:** 2025-02-13

**Authors:** Michaela Bártová, Alan Liška, Vendula Studená, Pavel Vojtíšek, Michal Kašpar, Tomáš Mikysek, Lenka Česlová, Ivan Švancara, Milan Sýs

**Affiliations:** 1 Department of Analytical Chemistry, Faculty of Chemical Technology, University of Pardubice, Studentská 573, 532 10 Pardubice, Czech Republic; michaela.bartova@student.upce.cz (M.B.); michal.kaspar@upce.cz (M.K.); tomas.mikysek@upce.cz (T.M.); lenka.ceslova@upce.cz (L.Č.); ivan.svancara@upce.cz (I.Š.); 2 J. Heyrovský Institute of Physical Chemistry of the CAS, Dolejškova 3, 182 23 Prague, Czech Republic; alan.liska@jh-inst.cas.cz; 3 Department of Inorganic Chemistry, Faculty of Sciences, Charles University, Albertov 2030, Prague 2, 120 00 Prague, Czech Republic; wendy.drak@seznam.cz (V.S.); pavojt@natur.cuni.cz (P.V.)

**Keywords:** dinuclear copper(II) complexes, biomimetic catalysis, oxidative transformation, organic dyes, pharmaceuticals

## Abstract

In this study, three dinuclear copper(II) complexes of ligand 2,6-bis[(*N*-methyl-piperazine-1-yl)methyl]-4-formyl phenol (L1) and one of 2,6-bis[(*N*-methylpiperazine-1-yl)methyl]-4-formyl phenol dimethylacetal (L2) with copper(II) ions have been investigated as new types of biomimetic catalysts for the oxidative transformation of different aminophenols and phenyldiamines. All the complexes of interest were newly synthesized and further characterized by IR spectroscopy, UV-Vis and mass spectrometry, X-ray diffraction, and selected electrochemical measurements. Crystal structures of these dinuclear copper(II) complexes have revealed that the coordination-shell geometry of copper atoms is close to a tetragonal pyramid. Catecholase, phenoxazinone synthase, and horseradish peroxidase-like activities were observed in pure methanol and water–methanol mixtures in the presence of molecular oxygen. The potential applicability of the complexes under study is discussed with respect to their possibilities and limitations in the replacement of natural copper-containing oxidoreductases in the oxidative degradation of water-insoluble chlorinated aminophenols in the dye industry or in the production of phenoxazine-based drugs.

## 1. Introduction

In general, various substituted aminophenols and phenylenediamines are widely used in the color dyeing process [1,2,3], drug synthesis [4], and the production of plastics as stabilizers or polymerization initiators [5]. In the presence of hydrogen peroxide as their oxidizer in an alkaline environment and with a coupling agent (dihydroxyphenols), these substances are subject to oxidative permanent dye formation [3]. The entire dying process is very complex, as it involves several consecutive chemical reactions that result in main yields, but also in numerous by-products [2].

In addition to their direct negative impact on human health [6], harmful compounds present in dye formulations along with their products of subsequent reactions pose a serious environmental risk. The most common dye ingredient, *p*-phenylenediamine (PPD), can also be found in dark henna temporary tattoos [7] and in rubber products, where it acts as an antioxidant. PPD and its oxidation products PPD-quinones are able to extensively pollute the ecosystem, thus establishing a novel group of environmental pollutants [8].

The above-mentioned aromatic organic compounds represent specific substrates for polyphenol oxidase enzymes, including horseradish peroxidase, tyrosinase, laccase, and phenoxazinone synthase. These biocatalysts can be considered among the most frequently mentioned metalloenzymes that are being investigated for drug synthesis [9,10] and environmentally friendly water treatment, especially in the decolorization of synthetic dyes [11,12,13], or even in the biodegradation of plastics [14]. These enzymes utilize molecular (di)oxygen as an electron acceptor to specifically catalyze the oxidation of a wide variety of organic substrates, such as phenols, ketones, phosphates, and amines, to the corresponding radicals [15,16].

In order to guarantee their long-term catalytic activity, scientists around the world are trying to replace naturally unstable enzymes with their synthetic analogs besides the nanostructured metal catalysts [17,18,19]. These so-called biomimetic enzymes also include a wide range of coordination compounds. Since tyrosinase, laccase, and phenoxazinone synthase have different numbers of copper atoms coordinated via histidine residues in their active centers, designers of catalytically active biomimetic systems usually try to replace histidines with artificially synthesized ligands [20]. Complexes with divalent copper, in which Cu(II)/Cu(I) redox couple plays a crucial role, include a specific group of these so-called artificial enzymes. Despite their high variability in the number of copper atoms and designed ligands, in organic solvents, most of them exhibit only catechol oxidase-like catalytic activity [20,21].

In this work, an earlier introduced dinuclear copper(II) complex [22,23,24] along with its three newly synthesized analogues based on 2,6-bis[(*N*-methylpiperazine-1-yl)methyl]-4-formyl phenol (L1) and one of 2,6-bis[(*N*-methylpiperazine-1-yl)methyl]-4-formyl phenol dimethylacetal (L2) ligands are investigated for their ability to mimic catechol oxidase, phenoxazinone synthase, and horseradish peroxidase catalytic activities towards selected substrates, such as dihydroxyphenols, aminophenols, and phenylenediamines, in pure methanol and methanolic-aqueous mixtures using a standard kinetic spectrophotometric method [25] and mass spectrometry. The main goal of the present study is to define the effect of their molecular structure on catalytic efficiency, thus revealing the character of a chemical bond between the central copper and oxygen atoms of bidentate ligands and the role of formyl or dimethyl acetal groups in the cationic ligands. The results have indicated that copper complexes under study exhibit a certain specificity to substrates that can donate their electron pair. It follows that these biomimetic catalysts can find their potential application in different branches of organic synthesis, especially in the production of pharmaceuticals and in the processing of organic dyes.

## 2. Results

### 2.1. Synthesis of Dinuclear Copper(II) Complexes

**Scheme 1:**
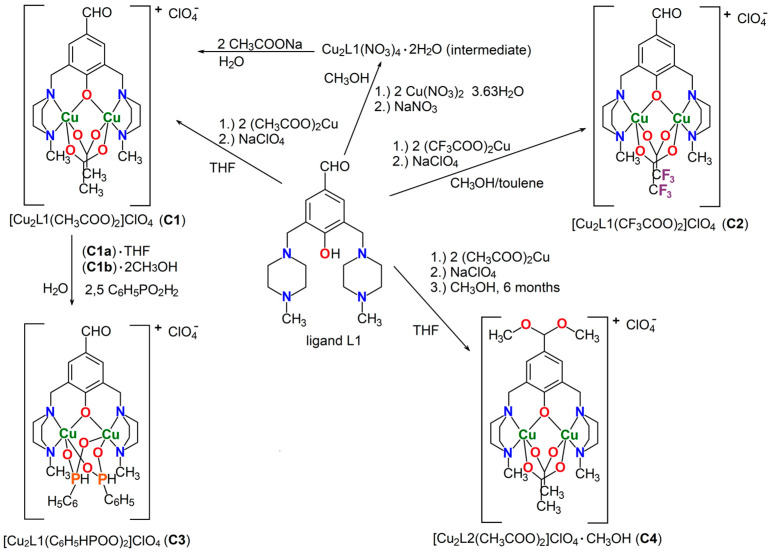
Synthesis of dinuclear copper(II) complexes C1–4.

Syntheses of complexes 1–4 are summarized in Scheme 1. As evident, they were synthesized using coordination reactions of ligand L1 with the corresponding divalent copper compounds, in which counter anions act as bidentate ligands. During the synthesis itself, certain specifics were revealed. Prolonged refluxing of C1 in dry MeOH for a couple of hours or prolonged standing for a couple of months at room temperature gave crystals of C4 containing a new ligand L2. To avoid the solvent effect resulting in the formation of the acetal product, an alternative procedure using tetrahydrofuran (THF) as solvent was chosen when a new compound solvated by THF (C1a) could be obtained. Interactions of the complex C1 with selected anions (phenylphosphinate, phenylphosphonate, and phenylboronate) were tested to show that only reaction with sodium phenylphosphonate in the presence of the corresponding acid led to the formation of a new complex C3 at room temperature.

### 2.2. Crystal Structures of Synthesized Dinuclear Copper(II) Complexes

**Figure 1:**
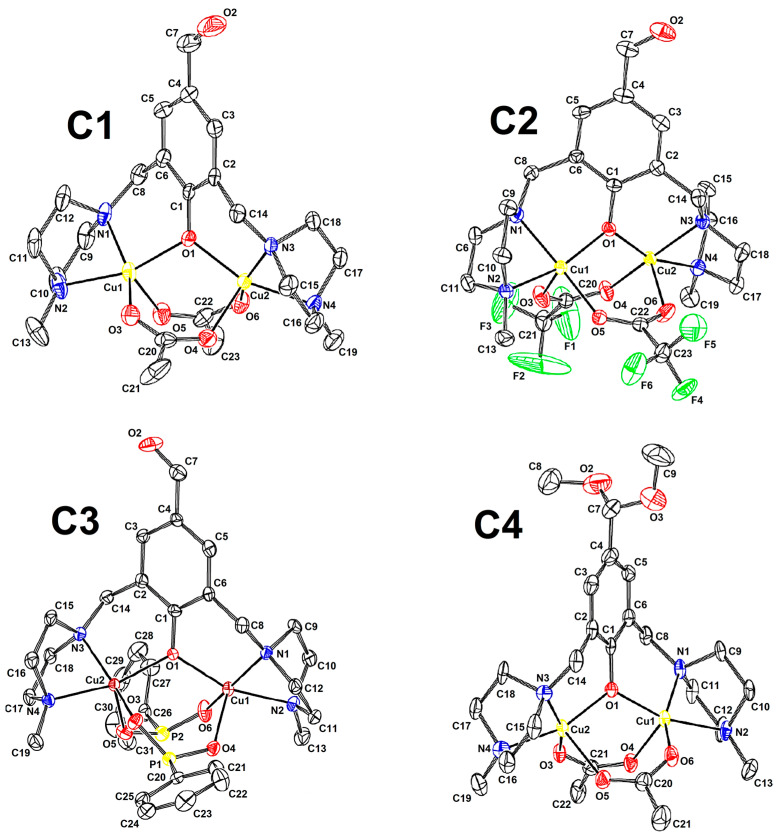
X-Ray crystal structures of [Cu_2_L1(CH_3_COO)_2_]ClO_4_ (C1), [Cu_2_L1(CF_3_COO)_2_(ClO_4_)] (C2), [Cu_2_L1(C_6_H_5_HPOO)_2_]ClO_4_ (C3), and [Cu_2_L2(CH_3_COO)_2_]ClO_4_ (C4) all without solvent molecule and coordinated perchlorate ion; all with thermal elipsoids at the 50% probability level. Note: In all the above-illustrated structures, hydrogen atoms are omitted for better clarity.

The structure of the [Cu_2_L1(CH_3_COO)_2_]ClO_4_ • THF complex (C1a) consists of a complex dinuclear cation, a perchlorate anion, and a non-coordinated solvent molecule. Copper atoms are connected to the ligand via the phenolate oxygen bridge, and two nitrogen atoms from both pendant arms. Two remaining coordination sites on each copper atom are used for “bridge bonding” of two acetate anions (Figure 1) when the coordination number (C.N.) of each Cu(II) is five. The shape of the coordination sphere is near to the tetragonal pyramid according to *τ* parameters [26] and the selected stereochemical parameters are given in Table 1. Further selected interatomic distances and angles are summarized in Supplementary Material (Table S2). The methanol solvatomorph, [Cu_2_L1(CH_3_COO)_2_]ClO_4_ • 2CH_3_OH complex (C1b) does not differ from its tetrahydrofuran analog in the crystal structure because the solvent molecules remain again out of the coordination sphere of Cu(II) nucleus.

Crystallographic analysis of complexes C1a and C1b indicates that they consist of two copper atoms Cu1 and Cu2, both as divalent cations and one non-coordinated perchlorate anion. On the other hand, the crystal structure of the [Cu_2_L1(CF_3_COO)_2_(ClO_4_)][Cu_2_L1(CF_3_COO)_2_]ClO_4_ complex (C2) comprises two different units that are fixed by *π*-*π* stacking between the benzene ring and the carbonyl group. The main stereochemical difference between these two units lies in C.N. of Cu(II); 6 + 5 in the neutral complex unit and 5 + 5 in the complex cation. The C.N. six is reached by the coordination of the perchlorate anion as a monodentate ligand (Figure 1). The interaction of the perchlorate ion is relatively weak due to the copper-oxygen distance of 2.833(4) Å. In the second moiety, coordination spheres of both copper(II) cations are formed by five bonds, analogically to the case of complexes C1a and C1b (Figure 1).

The crystal structure of [Cu_2_L1(C_6_H_5_HPOO)_2_]ClO_4_ complex (C3) is similar to the first complex C1 with the difference that the Cu-Cu distance is only longer by approximately 0.2 Å, probably due to the larger size of bridging phenylphosphinate anion in comparison with acetate ion. The structure of the complex cation is depicted in Figure 1. Stereochemical parameters of the Cu(II) shell are listed again in Table 1.

As evident from Figure 1, the crystal structure of [Cu_2_L2(CH_3_COO)_2_]ClO_4_ • CH_3_OH complex (C4) shows no differences in the coordination sphere of both Cu(II) ions from the complex C1, the C.N. units are five for both Cu(II) ions. Stereochemical parameters of the Cu(II) shell in Table 1 indicate that the replacement of the aldehyde group with acetal has no effect on the geometry of the coordination shell.

The parameter *τ*° = °|*α* − *β*|/60 (where *α* and *β* [°] illustrate the two largest angles in the coordination shell) was used to define the shape of the coordination polyhedron of the Cu(II) ions. The value *τ* = 0 corresponds to the ideal tetragonal pyramid (C_4v_) and the value *τ* = 1 corresponds to the ideal trigonal bipyramid (D_3h_). The coordination spheres of all synthetized dinuclear copper complexes (C1–4) are near a tetragonal pyramid with symmetry C_4v_ [26].

### 2.3. Ultraviolet-Visible Spectroscopy of Dinuclear Copper(II) Complexes

In pure MeOH, all four synthesized complexes displayed characteristic absorption bands at the following wavelength (*λ*): 285 nm for C1, 260 nm for C2, 281 nm for C3, and 278 nm for C4 (see Figure S1, belonging to Supplementary Materials). By determining the absorbance of these bands for varying concentrations from 50 to 300 μmol L^−1^, the values of the corresponding molar absorption coefficients (*ε*) were determined using the Lambert-Beer law, namely 2.4 × 10^4^, 1.3 × 10^4^, 1.6 × 10^4^, and 1.7 × 10^4^ L mol^−1^cm^−1^ for C1, C2, C3, and C4, respectively.

### 2.4. Infrared Spectroscopy of Dinuclear Copper(II) Complexes

Infrared spectra of ligand L1 (Figure S2) and its derived Cu(II) complexes (Figure S2–S5) were measured. Aldehyde valence vibrations bands are close to 1680 cm^−1^ *ν*_s_ (C=O) in all investigated complexes (range from 1661 cm^−1^ to 1702 cm^−1^), which corresponds to the value of free L1 (1679 cm^−1^); analogous valence vibration of 4-hydroxybenzaldehyde being observed at 1698 cm^−1^ [27]. Adsorption bands for *ν*_s_ (C-H) of the carbonyl group at ~2850–2940 cm^−1^ were also evident enough in the L1 spectrum.

Therefore, the aldehyde groups are not coordinated and participate in hydrogen bonds. Here, it is important to emphasize that the infrared spectra manifest of all the involved complexes (C1–3) did not overlap with the ligand L1 response.

The bands corresponding to uncoordinated perchlorate ions, namely those at 928 cm^−1^ *ν*_s_ (ClO), 460 cm^−1^ δ_s_ (ClO), 1120 cm^−1^ *ν*_as_ (ClO), 625 cm^−1^ δ_as_ (ClO) can be expected for salts containing anions [28]. A relatively simple pattern where the only split *ν*_3_ was found for complex C1, while a rather complicated system of bands was ascertained for complex C2, which indicates coordinated and uncoordinated types of perchlorate anions. Reliable assignments of coordinated acetate, trifluoroacetate, and trichloroacetate ions with expected bands below 1700 cm^−1^ are difficult or even impossible due to coincidence with ligand bands.

A complicated overlap of vibration bands of Cl-O and P-O was observed only in the infrared spectrum of [Cu_2_L1(C_6_H_5_HPOO)_2_]ClO_4_ complex (C3). The bands corresponding to the valence vibration P-O (approximately at 951.5 cm^−1^) were not discussed due to coincidence with bands of perchlorate. In addition, the less pronounced valence P-H vibration *ν*_1_ was distinguishable at 2320 cm^−1^ [29].

### 2.5. Mass Spectrometry of Dinuclear Copper(II) Complexes

All synthesized dinuclear copper(II) complexes were subjected to electrospray ionization mass spectrometry analysis in the positive ion mode by direct infusion into a QTRAP 4500 mass spectrometer from AB Sciex (Framingham, USA). From mass spectra (Figure S6), molecular peak values (*m*/*z*) of individual complexes were the following: 589 for C1, 697 for C2, 753 for C3, and 635 for C4. These values are in accordance with the corresponding molecular weights of their cationic ligands.

### 2.6. Electrochemical Properties of Dinuclear Copper(II) Complexes

**Figure 2:**
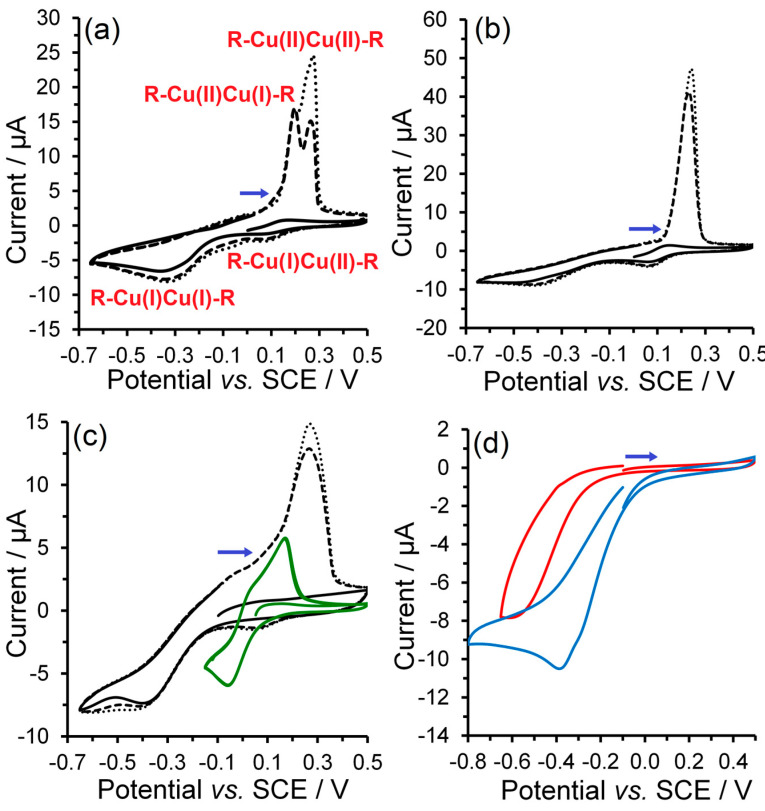
Repetitive cyclic voltammograms of 500 μmol L^−1^ C2 (**a**), C3 (**b**), and C4 with copper(II) acetate (green cycles; (**c**)) recorded on GCE in deaerated 99.99% (*v*/*v*) MeOH with 0.1 mol L^−1^ LiClO_4_ at potential step of 2.5 mV and scan rate of 50 mV s^−1^. Representative cyclic voltammograms of 500 μmol L^−1^ C4 (**d**) recorded on GCE in deaerated 99.99% (red) and 90% (*v*/*v*) acetonitrile (blue curve) with 0.1 mol L^−1^ LiClO_4_ at a potential step of 2.5 mV and scan rate of 50 mV s^−1^.

As already mentioned in the introduction, the electrochemical behavior of the Cu(II)/Cu(I) redox couple at the electrode could play a key role in the catalytic properties of biomimetic copper-based complexes [20,21]. For this reason, the electrochemical behavior of dinuclear copper(II) complexes had to be investigated using repetitive cyclic voltammetry of their 500 μmol L^−1^ deaerated solutions of methanol (MeOH) and acetonitrile (MeCN) with 0.1 mol L^−1^ LiClO_4_ content at bare glassy carbon electrode in the potential range from −0.8 to +0.5 V at scan rates of 50 mV s^−1^ (Figure 2). The electrochemical data obtained are gathered in Table S3.

These experiments have shown that all the investigated compounds (C1–4) follow similar electrochemical behavior with typical cyclic voltammograms reflecting the cathodic reduction in studied complexes transformed to R-Cu(II)Cu(I)-R within the first step, followed by the second reduction step to R-Cu(I)Cu(I)-R. The respective quasi-reversible two-step reoxidation was the most evident for C2, as depicted in the 2nd cycle in Figure 2a. For others, both reoxidation processes merged in the single (broad) anodic peak (see Figure 2b,c). These findings have agreed with our previous spectroelectrochemical study of C1 [24].

Furthermore, voltametric experiments with copper(II) acetate (Figure 2) proved that only bidentate ligands are released whereas copper atoms remain coordinated to the cationic ligand, confirming the stability of investigated complexes in pure organic solvents, as well as in the presence of 10% (*v*/*v*) water (Figure 2d). Moreover, copper(II) complexes exhibited an easier electrochemical reduction in Cu(II) to Cu(I) in aqueous-organic mixtures. However, this is not reflected in their higher catalytic activity in aqueous media, apparently, due to a competition for the active site between hydroxyl anions and amino groups in the molecules of substrate.

## 3. Discussion

### 3.1. Effect of Water on the Catalytic Activity of Dinuclear Copper(II) Complexes

All investigated copper(II) complexes showed maximum catecholase-like catalytic activity towards 3,5-DTBC in anhydrous polar organic solvents, such as acetonitrile, methanol, n-propanol, *N*,*N*-dimethylformamide, dimethylsulfoxide, and acetone. Unfortunately, as found in our previous study with the complex [Cu_2_L1(CH_3_COO)_2_]ClO_4_ [24], the presence of water had a negative effect on its catalytic efficiency. Even 5% water content (*v*/*v*) in MeOH caused a decrease in the values of the catalytic rate constant (38.8 h^−1^ for pure MeOH) by more than 70% (10.6 h^−1^ for 95% CH_3_OH). As can be expected, a similar decline in the catecholase-like activity towards 3,5-DTBC was also observed for the other copper(II) complexes investigated [24].

**Figure 3:**
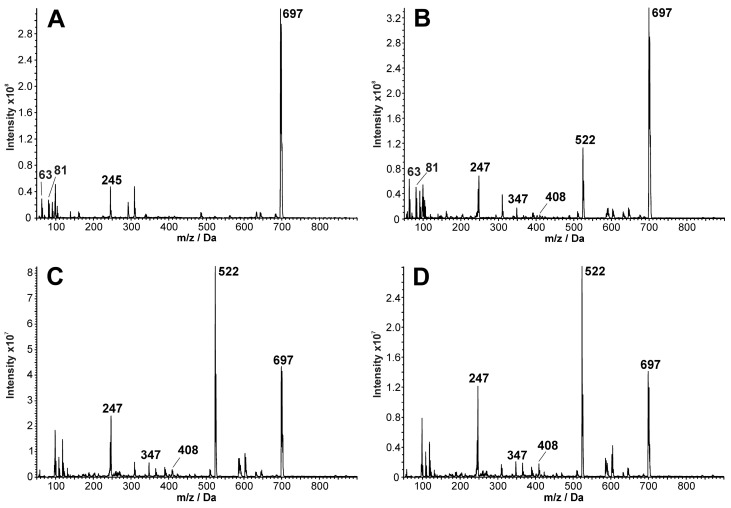
Positive-ion ESI mass spectra of compound C2 dissolved in pure (**A**), 90% (**B**), 70% (**C**), and 50% (*v*/*v*) MeCN (**D**).

In the present study, four dinuclear copper(II) complexes were investigated to understand the effect of water on their catalytic activity. As the technique of choice, electrospray ionization mass spectrometry (ESI-MS) was chosen and the proper analysis was performed using the direct infusion method with a series of solutions of the individual complexes in a mixture of MeCN and 0, 10, 30, and 50% (*v*/*v*) of water. From a comparison of their positive-ion tandem mass spectra, it can be concluded that the willingness to release one copper atom with the appropriate bidentate ligand increases with a higher water content. This phenomenon is demonstrated in Figure 3, being evident from the observation of a peak at a mass of 522 Da (*m*/*z*), which corresponds to the C2 cationic ligand that coordinates only one Cu atom with trifluoroacetate. Furthermore, a pair of peaks at 63 and 81 Da attributed to [^63^Cu]^+^ and [H_2_O + ^63^Cu]^+^ cations, respectively, indicate the dissociation of copper(II) trifluoroacetate.

It is also important to note that the peak at mass of 522 Da was not detected for C2 in pure MeCN (Figure 3A) nor in any corresponding fragmentation spectrum (Figure S7) unlike the peak at 247 Da which could be attributed to a mass of protonated L1 with neutral loss of *N*-methylpiperazine moiety. In addition, a decrease in the intensity of a peak at 697 Da [C2 cationic ligand] with a simultaneous increase in the peak at 522 Da confirms this statement. Finally, the analogical behavior was also observed for the other three complexes. This fact can be verified by comparing the MS spectra of **C3** in Figure S8, illustrating behavior between two peaks at 550 Da ([L1 + ^63^Cu + C_6_H_5_PHO_2_ + H]^+^) and 753 Da [C3 cationic ligand] depending on the water content in MeCN.

### 3.2. A Comprehensive Investigation of Biomimetic Catalytic Properties

Most of the copper complexes synthesized to date usually imitate natural enzyme active centers because they miss a protein part (apoenzyme) [30,31] responsible for the accurate recognition of the substrate. Consequently, multi-enzyme catalytic activity can be assumed for dinuclear copper(II) complexes that have to be comprehensively investigated using purposely selected substrates, as shown in the following (sub)sections. Partial results indicate that dinuclear copper(II) complexes catalyse reactions of aminophenols and phenyldiamines with dissolved oxygen giving rise to the corresponding oxidation products and water.

#### 3.2.1. Catechol Oxidase-Like Catalytic Activity of Dinuclear Copper(II) Complexes

Recently, a catechol oxidase-like activity has been proved for [Cu_2_L1(CH_3_COO)_2_]ClO_4_ complex (C1) that catalyzes the oxidation involving the dissolved oxygen and two molecules of substituted *ortho*-dihydroxyphenol when forming two molecules of water in one catalytic cycle [24]. Based on such a finding, other complexes (C2–4) were first subjected to the study of their biomimetic catechol oxidase activities and their catalytic performances of the air oxidation of 3,5-DTBC to 3,5-DTBQ investigated in pure MeOH when using UV-Vis absorption spectra.

**Figure 4:**
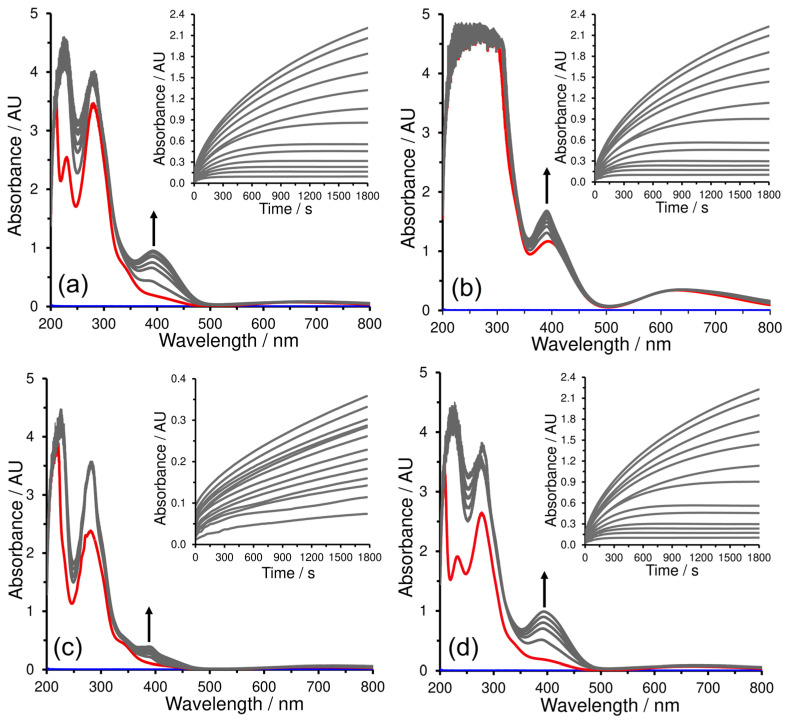
UV-Vis absorption spectra of 150 μmol L^−1^ solution of compound C1 (**a**), C3 (**c**), and C4 (**d**) or 750 μmol L^−1^ solution of compound C2 (**b**) (red spectra) with 500 μmol L^−1^ 3,5-DTBC in MeOH. The plots in inserts show kinetic curves obtained for the same concentrations of C1–4 catalyzing the oxidation of 0.05, 0.1, 0.15, 0.2, 0.3, 0.4, 0.6, 0.8, 1.0, 1.2, 1.5, 1.8, and 2.0 mmol L^−1^ 3,5-DTBC in MeOH at 400 nm for 30 min.

As evident from Figure 4 and the data surveyed in Table 2, dinuclear copper(II) complexes with two acetate groups in their molecular structure (C1 and C4) exhibit a comparable catechol oxidase-like activity, indicating that carbonyl and acetal functional groups have a similar impact on the strength of coordination bond between oxygen and copper atoms. An explanation of why these complexes based on copper(II) acetate (C1 and C4) catalyze the oxidation of 3,5-DTBC more effectively than those with copper (II) trifluoroacetate (C2) and phenylphosphonate (C3) can be in the different willingness of 3,5-DTBC to undergo deprotonation of hydroxy groups in a medium chosen, as well as different polarity of the bond between Cu-atoms and the respective anions. In the following sequence: C_2_H_3_O_2_^−^ (salt from weak organic acid) > C_6_H_5_O_3_P_2_^−^ (from medium-strong acid) > C_2_F_3_O_2_^−^ (from strong acid), the willingness to accept protons in the polar protic solvent decreases but, at the same time, the strength of their ionic bond with Cu-atoms increases, which is reflected in the highest value of $K_{m}$ and in the lowest maximum rate of catalysis for [Cu_2_L1(CF_3_COO)_2_]ClO_4_ complex (C2). The correlation between the values of catalytic rate constant and p*K*_A_ values of the corresponding organic acids (expressed as *r* = 0.9886) also confirms the behavior described above.

Regarding this, it was also confirmed that the addition of lithium acetate can markedly increase the catalytic efficiency of dinuclear copper(II) complexes. This is because the acetate acts like a base (proton acceptor) and facilitates the deprotonation of hydroxyl groups of the substrate [32].

#### 3.2.2. Phenoxazinone Synthase-Like Catalytic Activity of Dinuclear Copper(II) Complexes

**Figure 5:**
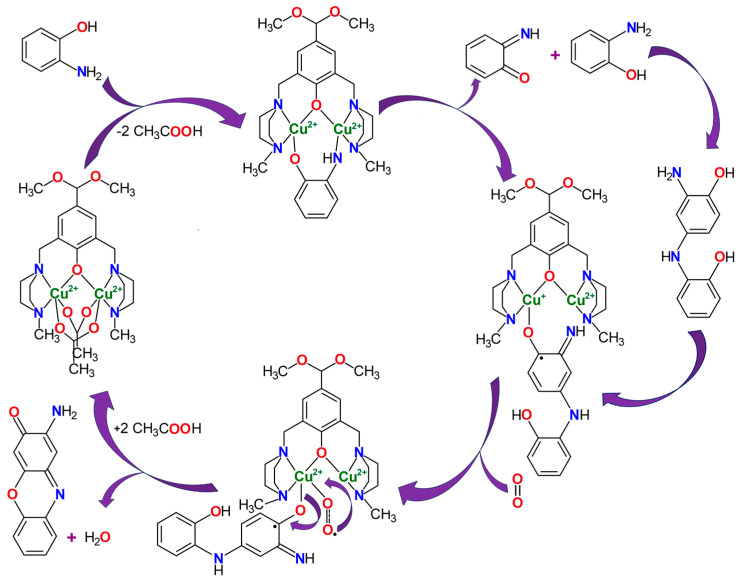
Proposed mechanism of catalytic activity of dinuclear copper(II) complex C4 toward the oxidation reaction of 2-aminophenol in the presence of oxygen, leading to 2-amino-3*H*-phenoxazine-3-one formation.

From the literature, it can be found that some mononuclear [33] and dinuclear [34] copper(II) complexes are able to catalyze an oxidative coupling of 2-aminophenol (2-AP) and its substituted analogs to form the corresponding phenoxazinone-based derivatives. For an easier understanding of phenoxazinone synthase-like catalytic activity, the proposed catalytic cycle of dinuclear copper(II) complex **C4** toward the oxidation reaction of 2-AP in the presence of oxygen, leading to 2-amino-3*H*-phenoxazine-3-one formation, is shown in Figure 5.

In order to gain new insights into the phenoxazinone synthase-like catalytic activity, [Cu_2_L1(CH_3_COO)_2_]ClO_4_ complex (C1) was selected for investigation in the oxidation of two selected *para*-aminophenols, namely 4-aminophenol and 4-amino-2-chlorophenol. In addition, the catalytically controlled oxidation of 2-aminophenol (2-AP) was investigated with naturally occurring dissolved oxygen, as well as artificially saturated oxygen.

Within the respective spectrophotometric study, it was found that dinuclear copper(II) complexes of 2,6-bis[(*N*-methylpiperazine-1-yl)methyl]-4-formyl phenol are able to catalyze the oxidation of *para*-aminophenols to the corresponding benzoquinone imines that subsequently react with substrate molecules (nucleophilic addition of amino group to *ortho* position). The resulting unstable intermediates undergo further oxidation with spontaneous cyclization and are transformed into phenoxazine-based derivatives [33], which is evident from UV-Vis spectra and from the increase in typical phenoxazinone chromophore bands at 430 nm (Figure S9a–b). As confirmation, comparable spectra were obtained for the oxidative conversion of 4-A2CP to 2-amino-8,10a-dichloro-10,10a-dihydro-3*H*-phenoxazin-3-one catalyzed by cobalt(III) octahedral complexes [35].

By comparing these spectral regions, it can be concluded that the 4-AP oxidation pathway catalyzed by dinuclear copper(II) complexes may not be unambiguous as that of 4-A2CP, which has one *ortho* position blocked. Almost three times higher $V_{max}^{app}$ value was calculated for 4-AP (0.56 AU s^−1^) than for 4-A2CP (0.21 AU s^−1^) using Lineweaver–Burk linearization (Figure S9c,d) suggesting that aminophenols substituted with electron withdrawing groups will be less degradable. On the other hand, the opposite phenomenon can be expected for substrates substituted with electron-donating groups, especially amines, which also function as proton acceptors and thus shift the reaction equilibrium in favor of the products. Either way, the complexes of interest can mimic phenoxazinone synthase-like activity also in the case of substituted *para*-aminophenols [36].

Furthermore, it was experimentally established that MeOH can contain ten times more dissolved molecular oxygen than water [37]. Typically, it is stated that at a temperature of 20 °C and a pressure of 1013 mbar, the dissolved oxygen content in MeOH can be approximately 40 mg L^−1^. If MeOH was saturated with oxygen from a pressure cylinder for 10 min, the turnover number of C1 was increased from 82 to 210 h^−1^ for 2-AP. In the opposite case, when MeOH was deareated with argon gas, there was a significant decrease in the limiting number of chemical conversions of substrate molecules to a value of 4.7 h^−1^. Everything indicates that with increasing molecular oxygen content in MeOH, the stability of the Cu(I)-amino-phenoxyl radical (Figure 5) decreases, which promotes faster conversion of 2-AP to the final product 2-amino-3*H*-phenoxazine-3-one.

#### 3.2.3. Horseradish Peroxidase-Like Catalytic Activity of Dinuclear Copper(II) Complexes

In addition to the aminophenols mentioned above, phenylenediamines (including some of their substituted analogs) are other coupling agents widely used for permanent dyeing processes [3]. Reactions of [Cu_2_L1(CH_3_COO)_2_]ClO_4_ complex (C1) with OPD or PPD in pure MeOH were monitored through UV-Vis spectrophotometry and liquid chromatography with photodiode array detection and tandem mass spectrometry (HPLC-PDA-MS) to confirm eventual horseradish peroxidase-like catalytic activity of related dinuclear copper(II) complexes.

**Figure 6:**
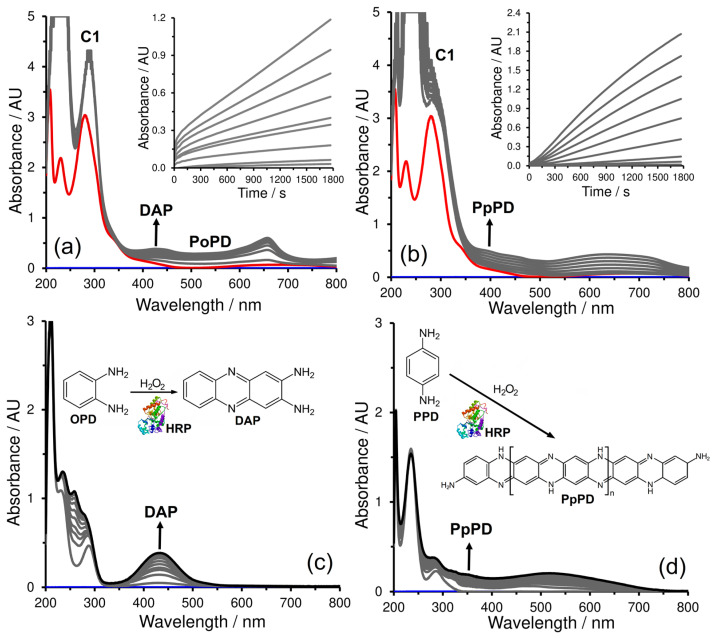
Spectral changes upon 500 μmol L^−1^ OPD (**a**) or PPD (**b**) catalyzed by 150 μmol L^−1^ compound C1 (red) in pure MeOH (blue spectrum). The inserted plots show kinetic curves obtained for the same concentration of C1 catalyzing the oxidation of 50, 150, 300, 600, 900, 1200, 1500, 1800, and 2100 µmolL^−1^ OPD (**a**) or PPD (**b**) in MeOH at 440 and 400 nm, respectively. Changes of the absorption spectrum of the reaction solution as a function of reaction time, with 15 μgmL^−1^ OPD (**c**) or 15 μgmL^−1^ PPD (**d**), 0.0002% H_2_O_2_, and 1.5 μgmL^−1^ HRP in 0.1 molL^−1^ phosphate buffer at pH 6.2 and 25 °C. The spectrum was recorded in intervals of 3 min after starting the reaction (up to 30 min; black spectra).

As demonstrated in Figure 6a, two characteristic absorption bands of forming 2,3-diaminophenazine (DAP) at 422 nm and PoPD at 663 nm were observed. For confirmation, the same absorption bands have already been found for these products [38,39,40,41,42]. Additional experiments proved that dinuclear copper(II) complexes are able to mimic horseradish peroxidase (HRP) activity (“provide identical oxidation products”) because the same band indicating DAP formation could also be found in the case of HRP (see Figure 6c). This happens without the need for hydrogen peroxide to be present.

The UV–Vis spectra of the PpPD formation displayed one well-recognizable absorption band at 400 nm (Figure 6b) belonging to the electronic transition of quinoneimine structures in the polymer chains [43,44]. Furthermore, a broad absorption band in the region from 550 to 750 nm can likely be attributed to PpPD with absorbed MeOH molecules [45]. For comparison, a similarly broad absorption band could also be observed in the case of using HRP in an aqueous solution of phosphate buffer, as evident in Figure 6d. The probability of the formation of this polymer was also supported by the results from HPLC-PDA-MS analysis, serving to confirm the catalytic oxidation of PPD to *p*-benzoquinone and its subsequent self-conjugation with PPD to a diphenylamine derivative (see Figure S10).

Since the oxidation of phenylenediamines with molecular oxygen does not result in well-defined products, it is only possible to compare the apparent values of their reaction kinetic parameters. This can be achieved if the dominant products are the polymers with similar absorption coefficients. The comparison of $K_{m}^{app}$ values, namely 0.7164 mmolL^−1^ OPD and 1.1557 mmolL^−1^ PPD, shows that substituted *ortho*-phenyl-diamines, as well as *ortho*-dihydroxyphenols [46], are more suitable substrates than their substituted *para*-analogs.

Based on these findings, it can be accepted that dinuclear copper(II) complexes catalyze the oxidation of OPD and PPD to form very reactive *o*-benzoquinone diimine and *p*-benzoquinone diimine, respectively, that both immediately undergo a coupling reaction with another substrate molecule, resulting, in some cases, in even more toxic intermediates [47]. Such quinoid products can also react in this way (via nucleophilic addition) with other substances of the mono- and di-phenol type, representing also environmental pollutants, which demonstrates the versatile applicability of copper(II) complexes in the degradation of environmental pollutants.

## 4. Materials and Methods

### 4.1. Chemicals and Reagents

The 2,6-bis[(*N*-methylpiperazine-1-yl)methyl]-4-formyl phenol (L1) was synthesized according to a procedure published in the original paper [26]. Copper(II) trifluoroacetate and trichloroacetate were prepared from copper(II) oxide and trifluoroacetic or trichloroacetic acid, respectively, and then characterized by elemental analysis. Glacial acetic acid, trichloroacetic acid, perchloric acid, and phenylphosphinic acid were obtained from Lach-Ner, s.r.o. (Neratovice, Czech Republic). Other chemicals employed in kinetic experiments, namely: 3,5-di-*tert*-butylcatechol (3,5-DTBC), 3,5-di-*tert*-butyl-*o*-benzoquinone (3,5-DTBQ), 4-aminophenol (4-AP), 4-amino-2-chlorophenol (4-A2CP), 1,2-phenylenediamine (OPD), 1,4-phenylenediamine (PPD), anhydrous methanol, and ammonium acetate were purchased from Merck KGaA (Darmstadt, Germany). A deionized water was obtained using a Milli-Q^®^ deionization unit from Merck Millipore (Burlington, MA, USA).

### 4.2. Apparatus

The quality of all the prepared compounds was verified by standard procedures using the elemental analysis and NMR, while the latter technique was used to verify the structure of the L1 molecule. Copper(II) salts used in syntheses of complexes of interest were analyzed by titration with EDTA in an ammonia buffer, indicated with murexide.

^1^H NMR spectra were recorded on a Varian Unity INOVA 300 device (Bruker, s.r.o.; Brno, Czech Republic) with resonance frequency for ^1^H NMR spectra set at 299,941 MHz; all the measurements were performed at 25 °C.

Infrared spectra were obtained using a single-ray spectrometer, model FTIR Magna 760 (Nicolet CZ s.r.o., Prague, Czech Republic), operated via the DRIFTS method. All the IR spectra were acquired from 400 to 4000 cm^−1^ with a spectral resolution of 4 cm^−1^, taking and accumulating 128 scans during one measurement. Spectrophotometric measurements in the UV/Vis region were performed in a standard quartz cuvette (*l*_C_ = 1 cm; Fisher Scientific, Pardubice, Czech Republic) using a UV2450 spectrophotometer (Shimadzu; Kyoto, Japan) controlled via UV-Probe software version 2.3.

The electrochemical cell incorporated the glassy carbon electrode (GCE) with a disk of 3 mm in diameter (type 6.09395.014; Metrohm Česká republika, s.r.o.; Prague, Czech Republic) as the working electrode, saturated calomel electrode (SCE) with the salt bridge filled by methanolic solution of 0.1 mol L^−1^ LiClO_4_ as the reference, and a platinum rod as the auxiliary electrode. This electrode assembly was connected to potentiostat/galvanostat AUTOLAB model PGSTAT101 operated via software NOVA 1.11.0 (all from Metrohm company specified above).

Identification of by-products from catalyzed oxidation reactions was performed with the aid of HPLC, consisting of an Agilent system Infinity II (Agilent Technologies; Santa Clara, USA) and comprising a flexible pump, vial sampler, column thermostat, a photodiode array (PDA) detector, and a single quadrupole mass spectrometer. The chromatographic separation was carried out using a Kinetex C18 column (150 × 3 mm, 3 µm; Phenomenex, Torrance, USA). Mobile phase “A” contained 5 mmol L^−1^ ammonium acetate in deionized water and mobile phase “B” than pure methanol when both components were employed for the gradient elution programmed in the following sequences: 0 min—5% B; 15 min—90% B; 18 min—90% B. The flow rate of the mobile phase was set to 0.4 mL min^−1^, the column temperature at 30 °C, and the injection volume was 5 µL.

### 4.3. Synthesis and Characterization of Copper(II) Complexes

#### 4.3.1. Synthesis of [Cu_2_L1(CH_3_COO)_2_]ClO_4_ (Complex C1)

Copper(II) acetate monohydrate (0.20 g; 1.0 mmol) was partly dissolved in tetrahydrofurane (THF; 30 mL), then, 5 mL THF solution of the ligand L1 (0.17 g; 0.5 mmol) was added. The resulting dark green solution was made turbid after the addition of 2 mL THF solution of sodium perchlorate (0.06 g; 0.5 mmol), and the reaction mixture was left overnight when the small dark green crystals formed were separated by filtration and dried in a desiccator for 3 days (0.22 g; 65 % yield). The elemental analysis gave these results: C, 38.62; H, 4.74; N, 7.10; Cu, 15.09. Calc. for C_23_H_35_N_4_O_10_ClCu_2_: C, 40.03; H, 5.08; N, 8.12; Cu, 18.42. Calc. for C_23_H_35_N_4_O_10_ClCu_2_·THF: C, 42.55; H, 5.65; N, 7.35; Cu, 16.69%. IR: 3432, 2879, 1682, 1601, 1469, 1407, 1361, 1341, 1305, 1289, 1224, 1181, 1146, 1093, 1008, 964, 935, 904, 812, 779, 725, 667, 624, 588, 569, 521, 466 cm^−1^; UV-Vis (H_2_O): 312, 235, 204; (CH_3_OH): 283, 229, 205 nm.

The [Cu_2_L1(CH_3_COO)_2_]ClO_4_ complex was also synthesized using another protocol, where copper(II) acetate monohydrate (0.20 g; 1.0 mmol) was suspended in dry methanol (5 mL). The mixture was refluxed together with a molecular sieve (under a drying tube) for 30 min. Then, the ligand L1 solution in dry methanol (0.17 g in 10 mL; 0.5 mmol) was added and the reflux continued for the next 2 h. After that, a solution with sodium perchlorate in dry methanol (0.06 g in 2.5 mL; 0.5 mmol) was added and this mixture was refluxed for 1 h. The resulting solution was kept at room temperature overnight. Then, the molecular sieve was removed, the solution evaporated to 2/3 of the original volume, the concentrate was replaced into a closed flask, left to rest overnight, and the next day cooled down in the fridge. After 2 h, long dark green needle crystals formed were isolated by filtrating and dried in a desiccator (over KOH) for 2 days (0.19 g; 55% yield).

The [Cu_2_L1(CH_3_COO)_2_]ClO_4_ complex could also be synthesized in two steps via intermediate Cu_2_L1(NO_3_)_4_ • 2H_2_O being prepared as follows. Copper(II) nitrate (3.63 hydrate) (126.4 mg; 0.5 mmol) was dissolved in 10 mL CH_3_OH and the solution of ligand L1 in methanol (86.6 mg in 4 mL; 0.25 mmol) was added. The resulting mixture was kept closed overnight and the next day the solution of sodium nitrate in methanol (21.3 mg in 2 mL; 0.25 mmol) was added. After slight stirring and gentle heating with a heating gun, a green solution was formed, kept in an open flask at room temperature for three days, and then placed in the fridge for one day. The dark green solid was filtrated and dried in a desiccator over KOH for 3 days (0.11 g; 58% yield). The elemental analysis: C, 30.04; H, 4.13; N, 14.72; Cu, 15.93; C:H:N:Cu = 9.99:16.5:3.96:1. Calc. for C_19_H_32_N_8_O_14_Cu_2_ • 2H_2_O: C, 30.10; H, 4.75; N, 14.78; Cu, 16.78 %. IR: 3448, 3034, 2757, 1671, 1601, 1483, 1449, 1383, 1364, 1289, 1191, 1152, 1104, 1062, 1012, 981, 900, 825, 810, 789, 759, 737, 680, 661, 632, 597, 496, 462 cm^−1^. UV-Vis (H_2_O): 312 nm.

#### 4.3.2. Synthesis of [Cu_2_L1(CF_3_COO)_2_(ClO_4_)][Cu_2_L1(CF_3_COO)_2_]ClO_4_ (C2)

Copper(II) trifluoroacetate dihydrate (162.8 mg; 0.5 mmol) was dissolved in methanol (8 mL); then, the solution of ligand L1 in methanol (86.6 mg in 2 mL; 0.25 mmol) plus the solution of sodium perchlorate in methanol (30.6 mg in 1 mL; 0.25 mmol) was added. The mixture was diluted with 2.2 mL toluene and kept in a closed flask overnight at room temperature. Afterward, the flask was opened and when ca. 3/4 of the original volume evaporated, the first crystals appeared at the bottom of the container. Herein, it is necessary to mention that a needle-shaped solid may appear on the wall of the flask and, in such a case, it is necessary to scratch these crystals back to the reaction mixture and stir it when the evaporation occurs rapidly. Otherwise, the dark green product was filtrated and dried in a desiccator for 2 days (125.6 mg; 63% yield). IR-analysis: 2880, 1702, 1605, 1578, 1487, 1471, 1449, 1328, 1315, 1305, 1272, 1208, 1187, 1147, 1103, 1082, 1040, 1011, 967, 896, 943, 812, 795, 781, 723, 623, 592, 570, 522, 473 cm^−1^. UV-Vis (H_2_O): 312, 235, 205 nm.

#### 4.3.3. Synthesis of [Cu_2_L1(C_6_H_5_HPOO)_2_]ClO_4_ (C3)

The previously prepared complex [Cu_2_L1(CH_3_COO)_2_]ClO_4_ (100 mg; 0.145 mmol) was dissolved in water (10 mL). Then, the solution of phenylphosphinic acid in water (51.5 mg in 4 mL; 0.363 mmol) was added when the solid acid had to be dissolved under heating. The mixture was kept in the flask overnight and then, the solution was evaporated in order to decrease its volume down to ca. 1/5 compared to the original amount. After that, the solution was kept in an open flask for 7 days. Then, the dark green crystals were isolated, dried between paper sheets and consequently in the desiccator (50.2 mg; 41% yield). IR-analysis: 3483, 2885, 2324, 1680, 1604, 1576, 1469, 1447, 1437, 1386, 1342, 1302, 1274, 1226, 1175, 1148, 1135, 1101, 1088, 1055, 1047, 1023, 1005, 982, 900, 811, 781, 746, 709, 692, 624, 582, 547, 486, 458 cm^−1^.

The synthesis of [Cu_2_L1(C_6_H_5_HPOO)_2_]ClO_4_ complex was simplified. This complex denoted as **C1** was dissolved in water as a solid (10.1 mg in 2 mL; 0.015 mmol) and then the solution of phenylphosphinic acid in water (5.2 mg in 0.5 mL; 0.036 mmol) together with the solution of sodium hydroxide in water (1.5 mg in 0.5 mL; 0.036 mmol) were added. Both solutions were mixed prior to adding to the complex solution. The mixture was stirred at room temperature for 30 min and then evaporated under the same conditions. After 34 days, dark green crystals were obtained. X-Ray analysis: crystal system: triclinic, *a* = 12.525(16) Å, *b* = 12.592(17) Å, *c* = 12.890(18) Å, *α* = 87.56(3)°, *β* = 87.82(3)°, *γ* = 68.09(3)°, *U* = 1884(5) Å^3^. The identity of the compound obtained by both procedures was proved by single crystal X-ray diffraction, number of reflections: 82.

#### 4.3.4. Synthesis of [Cu_2_L2(CH_3_COO)_2_]ClO_4_ (C4)

The reaction mixture in the preparation of complex **C1** within procedure No. 2 was partly evaporated and pure methanol was added in an amount like the original volume. After 6 months at room temperature, a solid product crystallized. The main crop of the product was characterized by X-ray powder diffraction: crystal system: orthorhombic, *a* = 19.59(12) Å, *b* = 14.59(9) Å, *c* = 23.87(14) Å, *α* = 90°, *β* = 90.6(3)°, *γ* = 90°, *U* = 6820(30) Å^3^, number of reflections: 45.

### 4.4. X-Ray Crystallographic Study

The diffraction-quality single crystals of the compounds were obtained directly from the reaction mixture solutions. The single crystals studied were transferred from the mother liquor to perfluoropolyalkyl ether and mounted on glass fibers. At the same temperature, the diffraction data were obtained using the Nonius Kappa CCD image plate from Bruker, s.r.o. (Brno, Czech Republic) in a diffractometer at 150 K when using a cryostream cooler from Oxford Cryosystem Ltd. (Long Hanborough, UK). The graphite monochromated MoKα radiation (*λ* = 0.71073 Å) was used and the obtained data was processed by APEX2 software from Bruker AXS Inc. (Madison, USA). The phase problem was solved by direct methods (SHELXS) [48]. To define the crystal structure, the software SHELXL97 was used [49]. Hydrogen atoms bonded to carbon atoms were located in calculated positions with isotropic temperature factors equal to 1.2-fold (or 1.5-fold) value of their neighboring atomic weights. The pictures of crystal structures were imaged with the aid of the Platon software [50]. For completeness, basic crystallographic data are surveyed in Table S1 (see Supplementary Materials).

### 4.5. Spectrophotometric Investigation of Biomimetic Oxidation Catalysis

#### 4.5.1. Catalytic Oxidation of 3,5-Di-*Tert*-Butylcatechol

The synthesized copper(II) complexes were examined for catechol oxidase-like activity towards 3,5-DTBC in pure MeOH by reactions of 150 μmol L^−1^ of the complex C1, C3, and C4, or 750 μmol L^−1^ C2; all being monitored for 30 min or 120 min, respectively, via the increase in the absorption band of the forming product of 3,5-DTBQ at 400 nm. Due to the significantly lower catalytic activity of complex C2, up to five times higher concentration of 3,5-DTBC had to be used.

To calculate the kinetic parameters, a calibration measurement of 10–100 μmol L^−1^ 3,5-DTBQ in pure MeOH at 400 nm had to be performed. For coefficients of determination (*R*^2^) higher than 0.9995, the obtained kinetic curves were properly fitted by the cubic functions (*f*(*x*) = *a*_3_ × *x*^3^ + *a*_2_*x*^2^ + *a*_1_*x* + *a*_0_), characterizing the 3,5-DTBQ formation (*c*) in the course of time (*t*). The subtracted *a*_1_ values, representing the reaction velocities ($V_{0}$), were subsequently used for the construction of the corresponding Lineweaver–Burk plots and for the calculation of kinetic parameters.

#### 4.5.2. Oxidation of *p*-Aminophenols Catalysed by C1

The phenoxazinone synthase-like activity was carried out by the reaction of 50–2100 μmol L^−1^ 4-AP or 4-A2CP with 150 μmol L^−1^ of the complex C1 in pure MeOH. The kinetics of the aerobic oxidation of these *p*-aminophenols catalyzed by C1 was investigated by monitoring the increase in absorbance as a function of time at 430 nm [51] when observing a characteristic absorption band of phenoxazine-based analogs in MeOH [36]. All the kinetic measurements were carried out for a period of 30 min and the apparent reaction velocity ($V_{0}^{app}$) of each catalytic reaction subtracted from the slope of linear regression (*f*(*x*) = *a*_1_*x* + *a*_0_) of the dependence “absorbance (*A*) vs. time (*t*)”, while values of coefficient of determination (*R*^2^) had to be higher than 0.9990. Values of $V_{0}^{app}$ were then used for the determination of kinetic parameters, such as the Michaelis constant ($K_{m}^{app}$) and the apparent maximum reaction velocity ($V_{max}^{app}$).

#### 4.5.3. Oxidative Polymerisation of Phenylenediamines Catalysed by C1

The horseradish peroxidase-like activity of **C1** was investigated on the oxidative polymerization of phenylamines, namely 1,2-phenylenediamine (OPD) and 1,4-phenylenediamine (PPD). The working conditions chosen were the same as in the previous case, and the formation of poly(*o*-phenylenediamine) (PoPD) and of poly(*p*-phenylenediamine) (PpPD) monitored at 440 or 400 nm, respectively; both for 30 min. For coefficients of determination (*R*^2^) higher than 0.9995, the kinetic curves were properly fitted by the cubic functions (*f*(*x*) = *a*_3_*x*^3^ + *a*_2_*x*^2^ + *a*_1_*x* + *a*_0_), characterizing the change in the dependence “absorbance (*A*) vs. time (*t*)”, when the subtracted *a*_1_ values represented apparent reaction velocities ($V_{0}^{app}$).

### 4.6. Mass Spectrometric Study of Dinuclear Copper(II) Complexes

ESI mass spectra were measured on a QTRAP 4500 mass spectrometer (AB Sciex; Framingham, USA) operating in electrospray ionization mode. The copper(II) complexes were dissolved in acetonitrile or aqueous-acetonitrile mixtures and then analyzed by direct infusion at a flow rate of 7 μL min^−1^. Mass spectra were recorded in the range from 50 to 900 Da (*m*/*z*) in the positive-ion mode. The experimental and instrumental parameters were set as follows: curtain gas of 10 psi, collision gas–medium, ion source gas of 20 psi, ion spray voltage of 2000 V, entrance potential of 10 V, declustering potential of 100 V, collision cell exit potential 14 V, and for fragmentation collision energy of 45 V.

## 5. Conclusions

In the study presented above, recently synthesized dinuclear copper(II) complexes of ligand 2,6-bis[(*N*-methylpiperazine-1-yl)methyl]-4-formyl phenol (L1) and one of 2,6-bis[(*N*-methylpiperazine-1-yl)methyl]-4-formyl phenol dimethylacetal (L2) have been investigated as new types of catalysts for the oxidative transformation of 4-AP, 4-A2CP, OPD, and PPD. One of the main observations was that unlike CH_3_CO_2_^−^, CF_3_CO_2_^−^, and C_6_H_5_PHO_2_^−^ bidentate ligands, the replacement of the formyl group (L1) with a dimethyl acetal group (L2) had not led to any significant improvement in their catalytic activity. In the following sequence C_2_H_3_O_2_^−^ (salt from weak organic acid) > C_6_H_5_O_3_P_2_^−^ (from medium-strong acid) > C_2_F_3_O_2_^−^ (from strong acid), the willingness to accept protons in the polar protic organic solvent decreases, but—at the same—the strength of their ionic bond with copper atoms increases, which is manifested by a higher catecholase-like catalytic activity towards 3,5-DTBC, namely 37.8 > 6.4 > 1.3 h^−1^.

The obtained apparent values of kinetic parameters showed that dinuclear copper(II) complexes should be potentially able to catalyze the oxidation of various substituted *ortho*- and *para*-dihydroxybenzenes, aminophenols, and phenylenediamines, which are considered as significant pollutants originating from the dyeing process. Unfortunately, the presence of water markedly lowers their catalytic efficiency due to the release of the copper atom with the respective bidentate ligand from the original coordination bond.

It can be concluded that this study points to the fact that the synthesis of water-stable dinuclear copper(II) complexes represents a principal step for the future development of effective wastewater treatment agents. Until this problem is solved, artificial enzymes will not be able to find their application like other promising materials and systems [52,53]. Despite all this, such complexes can be applicable in organic synthesis, especially in the production of drugs. Among typical examples, one can name phenoxazines widely used in medical treatments as these compounds exhibit antiviral, anticancer, anti-Alzheimer’s, antidiabetic, and antibiotic effects.

## Figures and Tables

**Table 1:** Selected stereochemical parameters τ in dinuclear copper(II) complexes **C1–4**.

Compound	Cu-Cu (Å)		*α* (°)	*β* (°)	*τ*
[Cu_2_L1(CH_3_COO)_2_]ClO_4_ • THF (C1a) ^1^	3.2793(7)	Cu1	161.1(1)	149.8(1)	0.19
		Cu2	160.3(1)	160.0(2)	0.01
[Cu_2_L1(CH_3_COO)_2_]ClO_4_ • THF (C1a) ^2^	3.2671(7)	Cu51	162.2(2)	153.1(1)	0.15
		Cu52	160.7(1)	157.3(2)	0.06
[Cu_2_L1(CH_3_COO)_2_]ClO_4_ • 2CH_3_OH (C1b)	3.2715(7)	Cu1	163.1(2)	154.5(2)	0.14
		Cu2	162.1(2)	155.6(2)	0.11
[Cu_2_L1(CF_3_COO)_2_]ClO_4_ (C2) ^3^	3.3666(6)	Cu51	164.5(1)	164.4(1)	0.00
		Cu52	164.9(1)	160.9(1)	0.07
[Cu_2_L1(CF_3_COO)_2_(ClO_4_)] (C2) ^4^	3.3611(6)	Cu2	163.2(1)	161.3(1)	0.03
[Cu_2_L1(C_6_H_5_HPOO)_2_]ClO_4_ (C3)	3.4645(5)	Cu1	165.6(1)	154.2(1)	0.19
		Cu2	161.4(1)	158.5(1)	0.05
[Cu_2_L2(CH_3_COO)_2_]ClO_4_ • CH_3_OH (C4)	3.280(1)	Cu1	162.8(2)	154.8(2)	0.13
		Cu2	161.3(2)	158.9(2)	0.04

^1^ first molecule, ^2^ second molecule, ^3^ molecule without perchlorate anion, and ^4^ molecule with perchlorate anion.

**Table 2:** Comparison of dinuclear copper(II) complexes in catechol oxidase-like catalytic activity towards 3,5-DTBC.

Compound	$K_{m}$ (mmol L^−1^)	$V_{max}$ (μmol L^−1^ s^−1^)	$k_{cat}$ (h^−1^)
[Cu_2_L1(CH_3_COO)_2_]ClO_2_ (C1)	0.348 ± 0.015	1.61 ± 0.08	37.8 ± 1.9
[Cu_2_L1(CF_3_COO)_2_]ClO_4_ (C2)	1.203 ± 0.067	0.29 ± 0.01	1.3 ± 0.1
[Cu_2_L1(C_6_H_5_HPOO)_2_]ClO_4_ (C3)	0.256 ± 0.016	0.30 ± 0.02	6.4 ± 0.4
[Cu_2_L2(CH_3_COO)_2_]ClO_4_ (C4)	0.284 ± 0.013	1.59 ± 0.07	39.1 ± 2.0

Notes: Values given as confidence intervals *x̄* ± *st*_1-α_/$\sqrt{n}$, where *x̄* is the arithmetic mean, *s* the standard deviation, and *t*_1-α_ the critical value (3.182) of student’s *t*-distribution for *n* (4) repetitions (3 degrees of freedom) of each analysis at *α* = 0.05.

## Data Availability

All the relevant data are only provided in the present paper.

## Supplementary Materials

The following supporting information can be downloaded at: https://www.mdpi.com/article/10.3390/ijms26041603/s1, Table S1: Selected crystallographic data presented dinuclear copper(II) complexes; Table S2: Selected bonds for C1a; values in Å and °; Figure S1: UV-Vis spectra of the 150 μmol L^−1^ dinuclear copper(II) complexes recorded in pure MeOH in 1 cm path quartz cuvette using a UV2450 spectrophotometer; Figure S2: IR spectrum of the ligand L1.; Figure S3: IR spectrum of the complex [Cu2L1(CH_3_COO)_2_]ClO_4_ (C1); Figure S4: IR spectrum of the complex [Cu_2_L1(CF_3_COO)_2_ClO_4_][Cu_2_L1(CF_3_COO)_2_]ClO_4_ (C2); Figure S5: IR spectrum of the complex [Cu_2_L1(C_6_H_5_HPO_2_)_2_]ClO_4_ (C3); Figure S6: Positive-ion ESI mass spectra of compounds C1 (A), C2 (B), C3 (C), and C4 (D) dissolved in pure MeCN; Table S3: Electrochemical behaviour of studied dinuclear copper(II) complexes at GCE in pure MeOH; Figure S7: Positive-ion tandem mass spectra of cationic part of compound C2 (*m*/*z* 697 Da) (A) and cationic part of compound C3 (*m*/*z* 753 Da) with neutral losses explanation (B); Figure S8: Positive-ion ESI mass spectra of compound C3 dissolved in pure (A), 90% (B), 70% (C), and 50% (*v*/*v*) MeCN (D); Figure S9: Spectral changes upon 500 μmol L^−1^ 4-AP (a) or 4-A2CP (b) catalysed by 150 μmol L^−1^ compound C1 (red) in pure MeOH (blue spectrum). Figure S10: HPLC chromatogram of the reaction mixture obtained from the oxidation of PPD catalyzed by compound C1 after 12 hours.

## Abbreviations

2-AP	2-aminophenol
4-AP	4-aminophenol
4-A2CP	4-amino-2-chlorophenol
3,5-DTBC	3,5-di-*tert*-butylcatechol
3,5-DTBQ	3,5-di-*tert*-butyl-*o*-benzoquinone
ESI-MS	electrospray ionisation mass spectrometry
GCE	glassy carbon electrode
HPLC-PDA-MS	liquid chromatography with photodiode array detection and tandem mass spectrometry
OPD	1,2-phenylenediamine
PoPD	poly(*o*-phenylenediamine)
PPD	1,4-phenylenediamine
PpPD	poly(*p*-phenylenediamine)
