# Supplementary material for: Dinuclear Copper(II) Complexes of 2,6-Bis[(N-Methylpiperazine-1-yl)methyl]-4-Formyl Phenol Ligand: Promising Biomimetic Catalysts for Dye Residue Degradation and Drug Synthesis
Source: Int J Mol Sci. 2025 Feb 13;26(4):1603. doi: 10.3390/ijms26041603 (PMC11855269; doi:10.3390/ijms26041603)
Supplement: Supplementary file 1 [file ijms-26-01603-s001.zip › ijms-3464407-supplementary.pdf]

Supplementary Materilas

Dinuclear copper(II) complexes of 2,6-bis[(*N*-methylpiperazine-1-yl)methyl]-4-formyl phenol ligand: Promising biomimetic catalysts for dye residue degradation and drug synthesis

Michaela Bártová [1], Alan Liška [2], Vendula Studená [3], Pavel Vojtíšek [3], Michal Kašpar [1], Tomáš Mikysek [1], Lenka Česlová [1], Ivan Švancara [1], and Milan Sýs [1,*]

[1] Department of Analytical Chemistry, Faculty of Chemical Technology, University of Pardubice, Studentská 573, 532 10 Pardubice, Czech Republic; michaela.bartova@student.upce.cz (M.B.); michal.kaspar@upce.cz (M.K.); tomas.mikysek@upce.cz (T.M.); lenka.ceslova@upce.cz (L.C.); ivan.svancara@upce.cz (I.S.); milan.sys@upce.cz (M.S.)
[2] J. Heyrovský Institute of Physical Chemistry of the CAS, Dolejškova 3, 18223 Prague, Czech Republic; Alan alan.liska@jh-inst.cas.cz (A.L.)
[3] Department of Inorganic Chemistry, Faculty of Sciences, Charles University, Albertov 2030, 120 00 Prague 2, Czech Republic; wendy.drak@seznam.cz (V.S); pavojt@natur.cuni.cz (P.V.)
* Correspondence: milan.sys@upce.cz; Tel.: +420-466-037-034

Contents

Basic crystallographic data (Table S1)
Selected bonds and angles (Tables S2)
IR spectra (Figures S1-S4)
Repetitive cyclic voltammograms of $Cu_2L1(C_6H_5HPOO)_2]ClO_4$ (Figure S5)
Values of peak separation (Table S3)
HPLC-PDA-MS analysis of PPD oxidation products (Figure S6)

Table S1. Selected crystallographic data presented dinuclear copper(II) complexes.

Compound label	**C1a**	**C1b**	**C2**	**C3**	**C4**
Formula					
Sum. formula	C27 H43 Cl Cu2 N4 O11	C24.60 H41.40 Cl Cu2 N4 O11.60	C46 H58 Cl2 Cu4 F12 N8 O20	C31 H47 Cl Cu2 N4 O13 P2	C26 H44 Cl Cu2 N4 O12
No. in CSD	1819061	1819062	1822011	1822434	1819063
Mr (g mol^{-1})	762.18	741.34	1596.06	908.19	767.18
Crystal dimension (mm)	0.654 × 0.430 × 0.175	0.653 × 0.431 × 0.140	0.557 × 0.146 × 0.145	0.231 × 0.134 × 0.080	0.83 × 0.19 × 0.09
Shape	prism	bar	prism	prism	plate
Color	green	blue	green	green	green
Crystal system	monoclinic	monoclinic	monoclinic	triclinic	orthorhombic
Space group H.-M.	P 21/c (no.14)	P 21/c (no.14)	P 21 (no.4)	P -1 (no.2)	P b c a (no.61)
Space group Hall	-P 2ybc	-P 2ybc	P 2yb	-P 1	-P 2ac 2ab
a (Å)	18.7848(7)	12.4627(9)	10.4788(3)	12.5095(4)	19.3899(13)
b (Å)	14.6462(6)	20.5247(15)	17.9182(6)	12.5572(4)	14.3985(10)
c (Å)	23.9508(8)	13.4923(10)	15.9881(4)	12.9268(4)	23.3777(16)
α (°)	90	90	90	88.0040(10)	90
β (°)	99.839(1)	112.282(2)	95.750(1)	87.9150(10)	90
γ (°)	90	90	90	68.2050(10)	90
U (Å^3)	6492.6(4)	3193.5(4)	2986.8(2)	1883.7(1)	6526.7(8)
Z	8	4	2	2	8
D_c (g.cm^{-3})	1.559	1.542	1.775	1.601	1.561
μ (mm^{-1})	1.455	1.478	1.611	1.353	1.450
Absorption correction	SADABS	SADABS	SADABS	SADABS	SADABS
F(000)	3168	1539	1616	940	3192
θ range of data (°)	1.726 - 26.000	1.909 - 24.010	1.280 - 27.499	1.577 - 26.057	1.965 - 24.999

	h −23 – 19	*h* −11 – 14	*h* −13 – 13	*h* −15 – 15	*h* −21 – 23
Index ranges	*k* −18 – 18	*k* −20 – 22	*k* −23 – 23	*k* −15 – 15	*k* −17 – 17
	l −29 – 29	*l* −15 – 14	*l* −20 – 19	*l* −15 – 15	*l* −27 – 27
Data complete (%)	99.9				
Number of reflections measured	62780	13505	39022	20605	71115
R_σ	0.0466	0.0597	0.0261	0.0614	0.0375
Number of reflections observed [$I > 2\sigma(I)$]	9197	3466	12719	5387	4383
Number of independent reflections	12751	4885	13670	7415	5517
R_{int}	0.0448	0.0404	0.0208	0.0396	0.0684
Data, restraints, parameters	12751; 0; 799	4885; 0; 403	13670; 7; 834	7415; 0; 486	5517; 0; 413
A, B [a]	0.0663; 22.677	0.0566; 4.7440	0.0367; 1.6721	0.0361; 0.7136	0.0324; 59.0241
Goodness of-fit on F^2	1.016	1.004	1.049	1.009	1.159
Final R,R' [$I \geq 2\sigma(I)$] [a,b]	0.0512; 0.1289	0.0430; 0.0997	0.0285; 0.0705	0.0381; 0.0750	0.0761; 0.1489
Max. shift/e.s.d.	0.001	0.000	0.001	0.000	0.000
Largest difference peak and hole (eÅ^3)	1.857; −1.087	1.216; −0.445	1.077; −0.665	0.588; −0.407	1.594; −0.823

[a] $w = 1/[\sigma^2(F_o^2) + (A * P)^2 + B * P]$, where $P = (F_o^2 + 2F_c^2)/3$

[b] $R = \Sigma|F_o - F_c|/\Sigma|F_c|$; $R' = [\Sigma w(F_o^2 - F_c^2)^2/\Sigma w(F_o^2)^2]^{1/2}$ (SHELXL-2014/7)

Table S2. Selected bonds for **C1a**; values in Å and °.

**Molecule 1, the geometry of dinuclear moiety**							
Cu1-O1	1.959(3)	O1-Cu1-N1	91.7(1)	Cu2-O1	1.960(3)	O1-Cu2-N3	92.0.(1)
Cu1-N1	2.020(3)	O1-Cu1-N2	162.2(1)	Cu2-N3	2.014(4)	O1-Cu2-N4	160.67(1)
Cu1-N2	2.038(4)	O1-Cu1-O3	95.8(1)	Cu2-N4	2.0514)	O1-Cu2-O4	94.4(1)
Cu1-O3	1.931(3)	O1-Cu1-O5	93.0(1)	Cu2-O4	2.145(3)	O1-Cu2-O6	95.6(2)
Cu1-O5	2.133(3)	N1-Cu1-N2	73.5(2)	Cu2-O6	1.922(3)	N3-Cu2-N4	73.6(2)
		N1-Cu1-O3	153.1(2)			N3-Cu2-O4	157.2(1)
C20-O3	1.275(6)	N1-Cu1-O5	102.1(1)	C22-O5	1.240(6)	N3-Cu2-O6	100.5(1)
C20-O4	1.240(6)	N2-Cu1-O3	93.7(2)	C22-O6	1.274(6)	N4-Cu2-O4	99.0(1)
O3-C20-O4	126.2(4)	N2-Cu1-O5	99.3(1)	O5-C22-O6	126.5(4)	N4-Cu2-O6	93.3(1)
		O3-Cu1-O5	103.3(1)			O5-Cu2-O6	101.7(1)
Cu1-Cu2	3.2793(7)			Cu1-O1-Cu2	113.6(1)		

**Molecule 2, the geometry of dinuclear moiety**							
Cu51-O51	1.956(3)	O51-Cu51-N51	92.3(1)	Cu52-O51	1.956(3)	O51-Cu52-N53	90.8(1)
Cu51-N51	2.008(4)	O51-Cu51-N52	160.3(1)	Cu52-N53	2.028(4)	O51-Cu52-N54	161.2(1)
Cu51-N52	2.039(4)	O51-Cu51-O53	92.0(1)	Cu52-N54	2.041(4)	O51-Cu52-O54	95.6(1)
Cu51-O53	2.173(3)	O51-Cu51-O55	96.5(1)	Cu52-O54	1.945(3)	O51-Cu52-O56	95.0(1)
Cu51-O55	1.919(3)	N51-Cu51-N52	73.9(1)	Cu52-O56	2.149(3)	N53-Cu52-N54	73.5(2)
		N51-Cu51-O53	96.0(1)			N53-Cu52-O54	149.8(1)
C70-O53	1.241(6)	N51-Cu51-O55	159.9(2)	C72-O55	1.267(5)	N53-Cu52-O56	101.1(1)
C70-O54	1.272(6)	N52-Cu51-O53	103.1(1)	C72-O56	1.235(5)	N54-Cu52-O54	93.4(2)
O53-C70-O54	125.3(4)	N52-Cu51-O55	92.7(1)	O55-C72-O56	126.4(4)	N54-Cu52-O56	98.2(1)
		O53-Cu51-O55	101.7(1)			O54-Cu52-O56	107.7(1)
Cu51-Cu52	3.2671(7)			Cu51-O51-Cu52	113.3(1)		

Selected bonds for **C1b**; values in Å and °

The geometry of dinuclear moiety							
Cu1-O1	1.959(3)	O1-Cu1-N1	92.3(1)	Cu2-O1	1.547(3)	O1-Cu2-N3	92.5(1)
Cu1-N1	2.007(4)	O1-Cu1-N2	162.1(1)	Cu2-N3	2.018(4)	O1-Cu2-N4	163.1(2)
Cu1-N2	2.055(4)	O1-Cu1-O3	96.2(1)	Cu2-N4	2.033(4)	O1-Cu2-O4	93.4(1)
Cu1-O3	1.925(3)	O1-Cu1-O5	94.7(1)	Cu2-O4	2.159(3)	O1-Cu2-O6	95.4(1)
Cu1-O5	2.164(3)	N1-Cu1-N2	73.6(2)	Cu2-O6	1.944(3)	N3-Cu2-N4	73.6(2)
		N1-Cu1-O3	155.5(1)			N3-Cu2-O4	100.8(1)
C20-O3	1.274(5)	N1-Cu1-O5	101.7(1)	C22-O5	1.262(5)	N3-Cu2-O6	154.5(1)
C20-O4	1.245(5)	N2-Cu1-O3	92.9(1)	C22-O6	1.270(6)	N4-Cu2-O4	93.7(2)
O3-C20-O4	125.9(4)	N2-Cu1-O5	98.9(1)	O5-C22-O6	124.8(4)	N4-Cu2-O6	102.1(1)
		O3-Cu1-O5	100..5(1)			O4-Cu2-O6	102.9(1)
Cu1-Cu2	3.2715(7)			Cu1-O1-Cu2	123.2(3)		

Selected bonds for **C2**; values in Å and °.

Molecule 1, the geometry of dinuclear moiety							
Cu1-O1	1.954(3)	O1-Cu1-N1	92.4(1)	Cu2-O1	1.957(3	O1-Cu2-N3	93.0(1)
Cu1-N1	2.023(3)	O1-Cu1-N2	164.6(1)	Cu2-N3	2.007(3)	O1-Cu2-N4	164.9(1)
Cu1-N2	2.020(3)	O1-Cu1-O3	99.9(1)	Cu2-N4	2.032(3)	O1-Cu2-O4	93.4(1)
Cu1-O3	1.939(3)	O1-Cu1-O5	88.2(1)	Cu2-O4	2.190(3)	O1-Cu2-O6	96.5(1)
Cu1-O5	2.260(3)	O1-Cu1-O11	77.7(1)	Cu2-O6	1.948(3)	N3-Cu2-N4	74.2(1)
Cu1-O11 ??	2.832(3)	N1-Cu1-N2	73.7(1)			N3-Cu2-O4	98.5(1)
		N1-Cu1-O3	164.8(1)	C23-O5	1.229(5)	N3-Cu2-O6	160.9(1)
C20-O3	1.241(5)	N1-Cu1-O5	93.1(1)	C23-O6	1.241(5)	N4-Cu2-O4	93.6(1)
C20-O4	1.235(5)	N1-Cu1-O11	93.0(1)	O5-C23-O6	131.4(4)	N4-Cu2-O6	93.5(1)
O3-C20-O4	130.8(4)	N2-Cu1-O3	92.8(1)			O4-Cu2-O6	97.5(1)
		N2-Cu1-O5	99.2(1)				
Cu1-Cu2	3.3666(6)	N2-Cu1-O11	95.9(1)	Cu1-O1-Cu2	118.8(1)		
		O3-Cu1-O5	96.7(1)				
		O3-Cu1-O11	80.4(1)				
		O5-Cu1-O11	164.7(1)				

Molecule 2, the geometry of dinuclear moiety

Cu51-O51	1.974(3)	O51-Cu51-N51	93.0(1)	Cu52-O51	1.967(3)	O51-Cu52-N53	93.3(1)
Cu51-N51	1.999(3)	O51-Cu51-N52	164.3(1)	Cu52-N53	1.994(3)	O51-Cu52-N54	161.3(1)
Cu51-N52	2.044(3)	O51-Cu51-O53	93.5(1)	Cu52-N54	2.045(3)	O51-Cu52-O54	99.0(1)
Cu51-O53	2.212(3)	O51-Cu51-O55	96.0(1)	Cu52-O54	1.927(3)	O51-Cu52-O56	89.4(1)
Cu51-O55	1.951(3)	N51-Cu51-N52	74.1(1)	Cu52-O56	2.247(3)	N53-Cu52-N54	74.1(1)
		N51-Cu51-O53	97.9(1)			N53-Cu52-O54	163.2(1)
C70-O53	1.223(5)	N51-Cu51-O55	161.7(1)	C72-O55	1.246(5)	N53-Cu52-O56	90.7(1)
C70-O54	1.248(5)	N52-Cu51-O53	97.0(1)	C72-O56	1.233(5)	N54-Cu52-O54	91.1(1)
O53-C70-O54	130.8(4)	N52-Cu51-O55	94.1(1)	O55-C72-O56	131.0(4)	N54-Cu52-O56	104.2(1)
		O53-Cu51-O55	97.4(1)			O54-Cu52-O56	100.7(1)
Cu51-Cu52	3.3611(6)			Cu51-O51-Cu52	117.0(1)		

Selected bonds for **C3**; values in Å and °.

**The geometry of dinuclear moiety**							
Cu1-O1	2.000(2)	O1-Cu1-N1	93.54(8)	Cu2-O1	1.983(2)	O1-Cu2-N3	93.45(9)
Cu1-N1	1.990(2)	O1-Cu1-N2	161.34(9)	Cu2-N3	1.993(2)	O1-Cu2-N4	165.59(9)
Cu1-N2	2.050(2)	O1-Cu1-O11	96.85(8)	Cu2-N4	2.037(2)	O1-Cu2-O12	92.87(9)
Cu1-O11	2.260(2)	O1-Cu1-O21	93.57(8)	Cu2-O12	1.925(2)	O1-Cu2-O22	95.58(8)
Cu1-O21	1.914(2)	N1-Cu1-N2	73.71(9)	Cu2-O22	2.194(2)	N3-Cu2-N4	74.2(1)
		N1-Cu1-O11	100.06(9)			N3-Cu2-O12	154.26(9)
P1-O11	1.503(2)	N1-Cu1-O21	158.5(1)	P2-O21	1.500(2)	N3-Cu2-O22	105.57(8)
P1-O12	1.512(2)	N2-Cu1-O11	98.75(9)	P2-O22	1.507(2)	N4-Cu2-O12	95.3(1)
O11-P1-O12	117.4(1)	N2-Cu1-O21	93.97(9)	O21-P2-O22	117.0(1)	N4-Cu2-O22	94.92(8)
		O11-Cu1-O21	99.25(9)			O12-Cu2-O22	98.61(8)
Cu1-Cu2	3.4645(5)			Cu1-O1-Cu2	120.9(1)		

Selected bonds for **C4**; values in Å and °.

**The geometry of dinuclear moiety**							
Cu1-O1	1.947(4)	O1-Cu1-N1	92.0(2)	Cu2-O1	1.941(4)	O1-Cu2-N3	91.9(2)
Cu1-N1	2.021(5)	O1-Cu1-N2	161.3(2)	Cu2-N3	2.017(5)	O1-Cu2-N4	162.7(2)
Cu1-N2	2.056(5)	O1-Cu1-O3	94.2(2)	Cu2-N4	2.036(5)	O1-Cu2-O4	96.9(2)
Cu1-O3	2.218(4)	O1-Cu1-O9	96.2(2)	Cu2-O4	1.853(5)	O1-Cu2-O10	93.8(2)
Cu1-O9	1.926(4)	N1-Cu1-N2	73.6(2)	Cu2-O10	2.137(5)	N3-Cu2-N4	73.6(2)
		N1-Cu1-O3	101.2(2)			N3-Cu2-O4	154.8(2)
C20-O3	1.261(8)	N1-Cu1-O9	158.9(2)	C22-O9	1.271(8)	N3-Cu2-O10	103.1(2)
C20-O4	1.259(8)	N2-Cu1-O3	100.2(2)	C22-O10	1.254(8)	N4-Cu2-O4	92.9(2)
O3-C20-O4	125.6(6)	N2-Cu1-O9	93.8(2)	O9-C22-O10	125.6(6)	N4-Cu2-O10	98.5(2)
		O3-Cu1-O9	97.6(2)			O4-Cu2-O10	99.8(2)
Cu1-Cu2	3.280(1)			Cu1-O1-Cu2	115.0(2)		

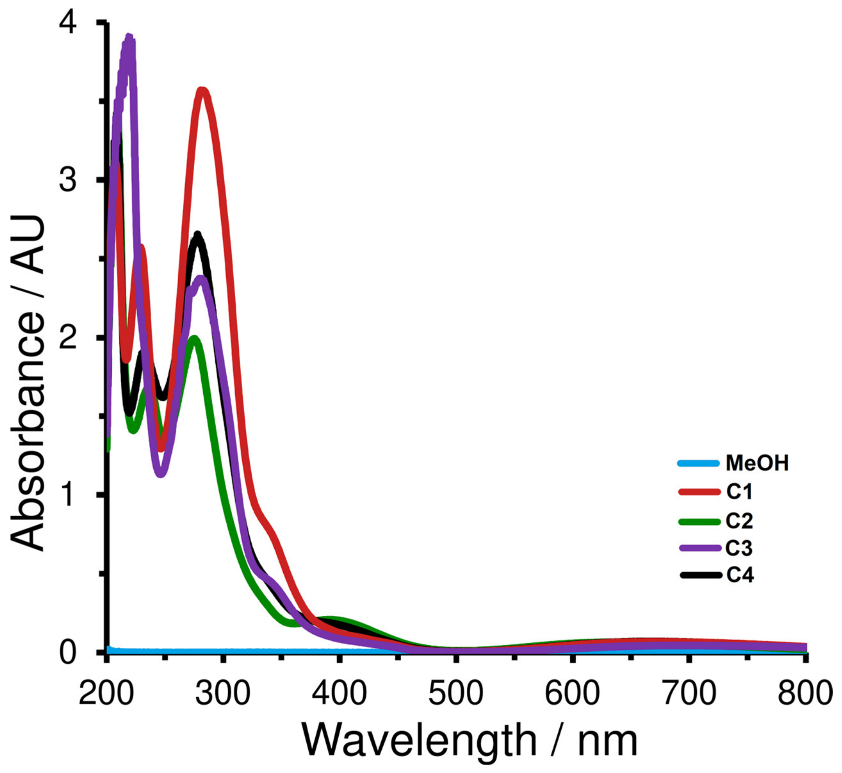

Figure S1. UV-Vis spectra of the 150 μmol L⁻¹ dinuclear copper(II) complexes recorded in pure MeOH in 1 cm path quartz cuvette using a UV2450 spectrophotometer.

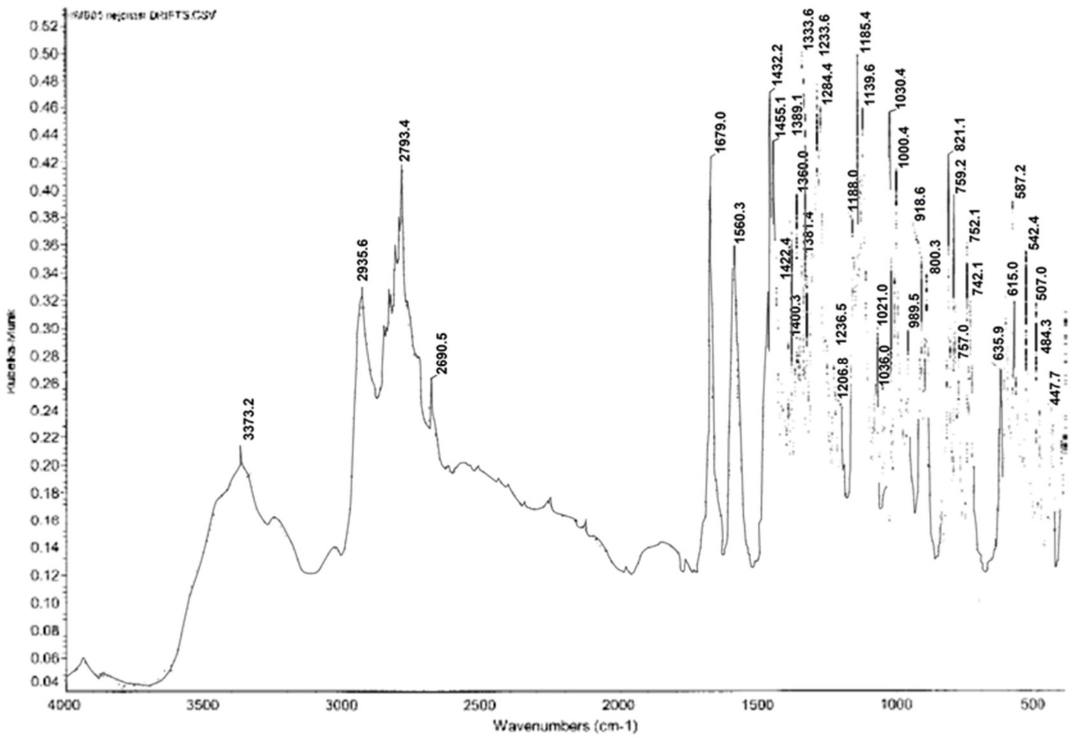

Figure S2. IR spectrum of the ligand L1.

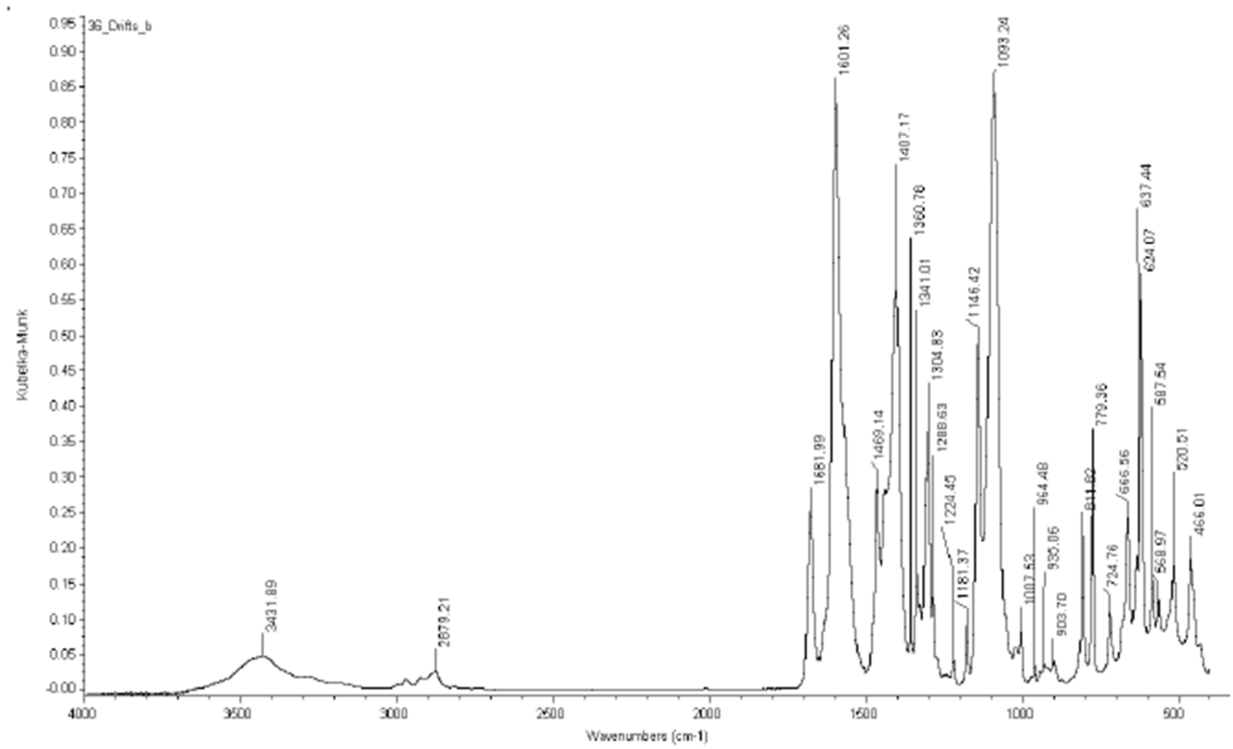

Figure S3. IR spectrum of the complex $[Cu_2L1(CH_3COO)_2]ClO_4$ (**C1**).

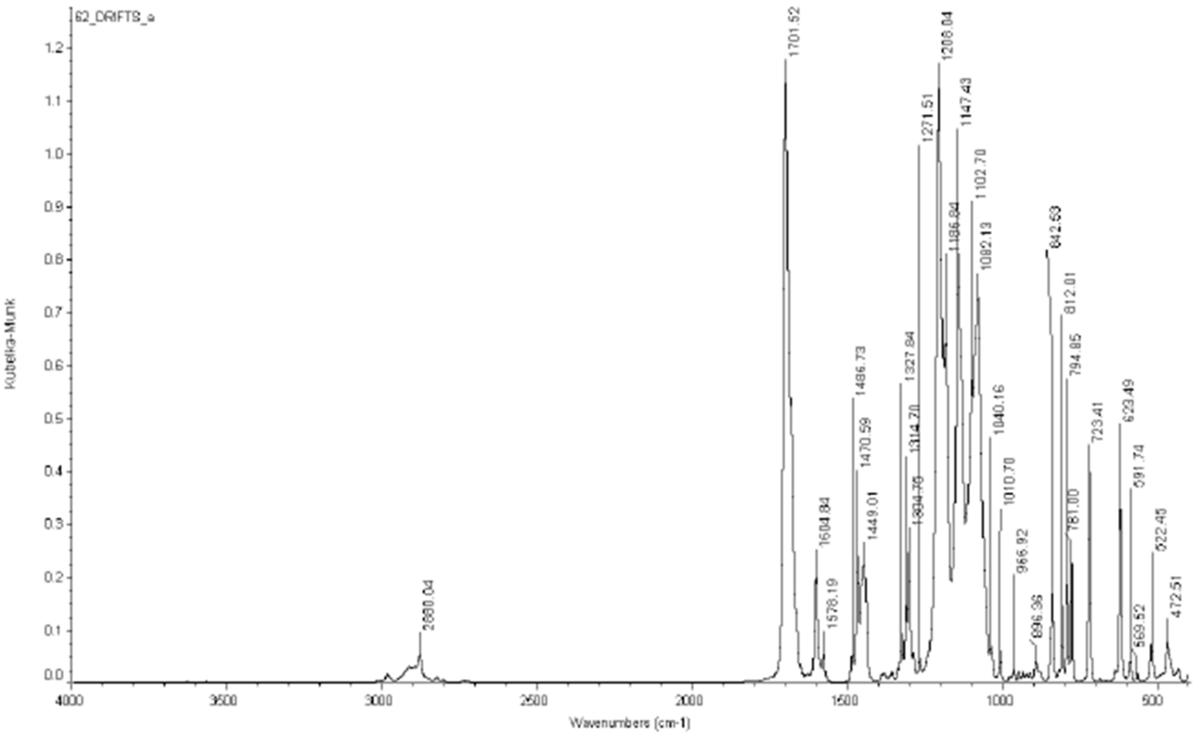

Figure S4. IR spectrum of the complex $[Cu_2L1(CF_3COO)_2ClO_4][Cu_2L1(CF_3COO)_2]ClO_4$ (**C2**).

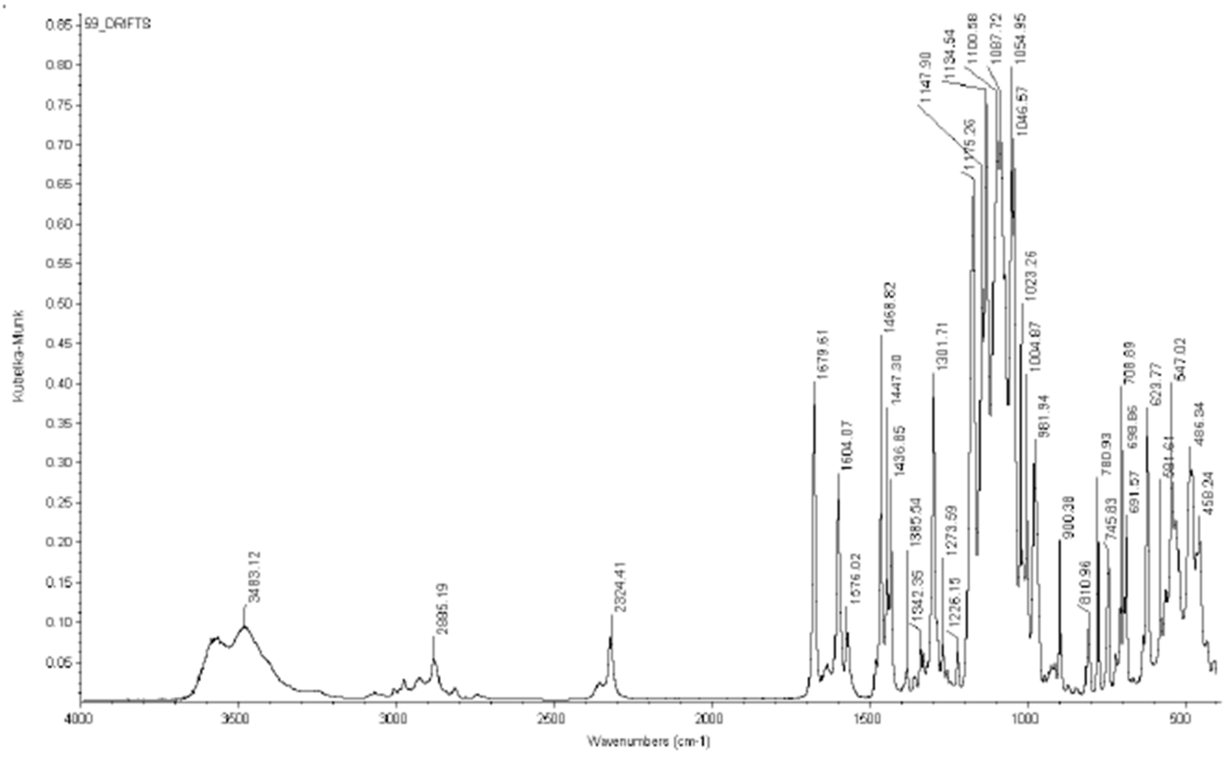

Figure S5. IR spectrum of the complex $[Cu_2L1(C_6H_5HPO_2)_2]ClO_4$ (**C3**).

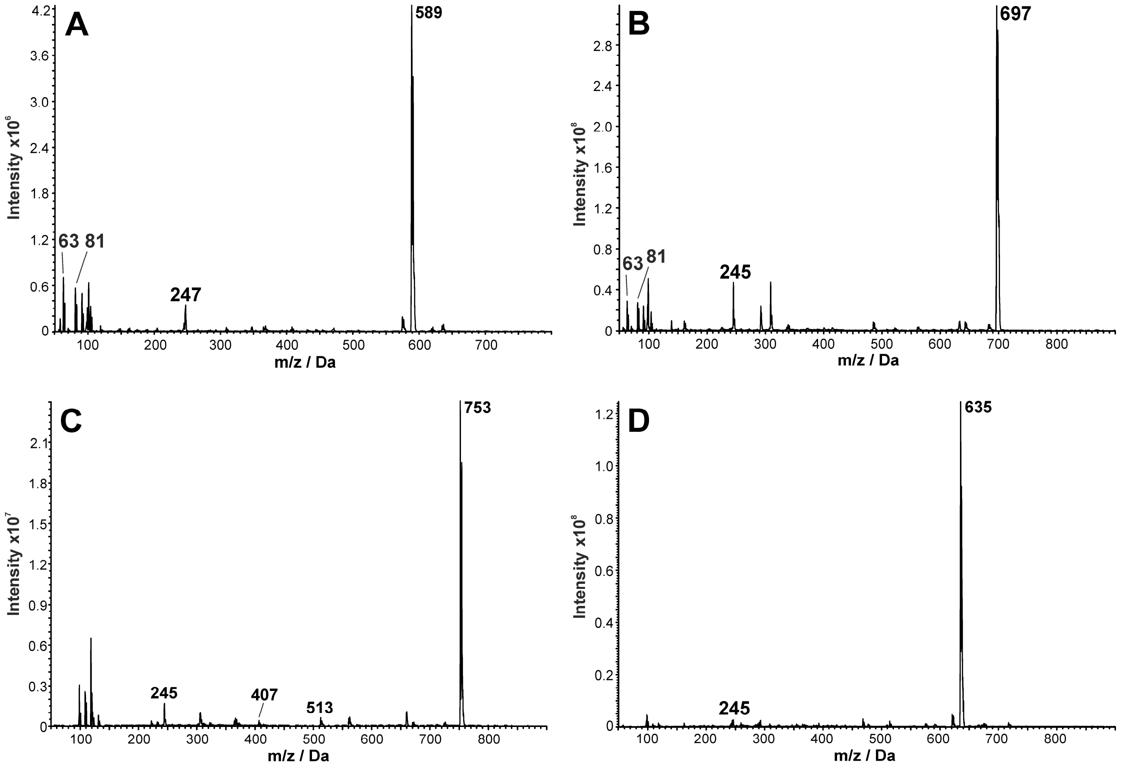

Figure S6. Positive-ion ESI mass spectra of compounds **C1** (A), **C2** (B), **C3** (C), and **C4** (D) dissolved in pure MeCN.

Table S3. Electrochemical behaviour of studied dinuclear copper(II) complexes at GCE in pure MeOH.

Compound	E_P^a (V)	E_P^c (V)	ΔE_P (V)
$[Cu_2L1(CH_3COO)_2]ClO_2$ (**C1**)	0.354	−0.550	0.904
$[Cu_2L1(CF_3COO)_2]ClO_4$ (**C2**)	0.259	−0.337	0.596
$[Cu_2L1(C_6H_5HPOO)_2]ClO_4$ (**C3**)	0.229	−0.417	0.646
$[Cu_2L2(CH_3COO)_2]ClO_4$ (**C4**)	0.264	−0.398	0.662

Notes: Values of E_P^a; anodic peak potential *vs.* SCE, E_P^c; cathodic peak potential *vs.* SCE, and ΔE_P; peak separation calculated for two-step reduction and the corresponding reoxidation.

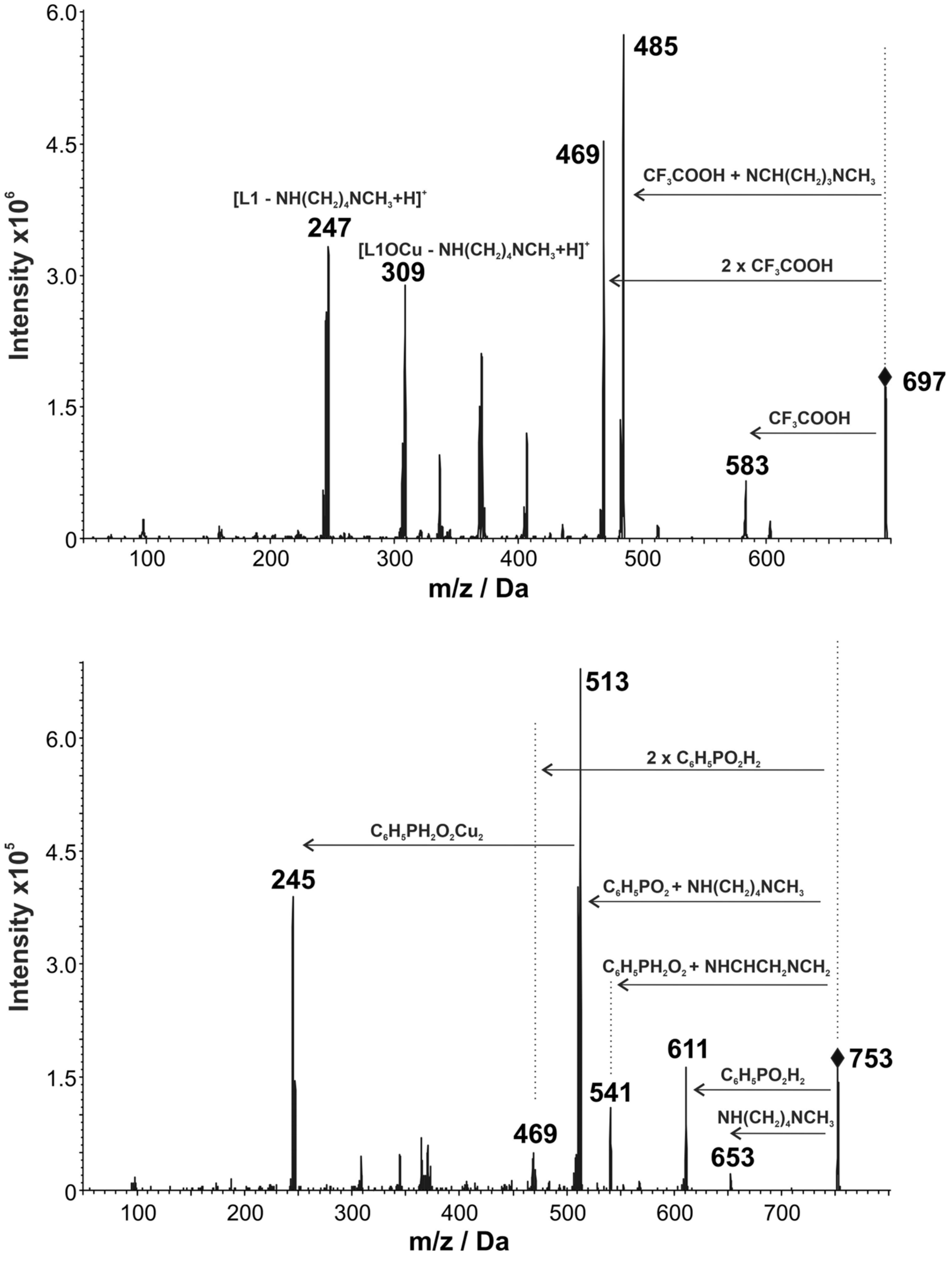

Figure S7. Positive-ion tandem mass spectra of cationic part of compound **C2** (*m*/*z* 697 Da) (A) and cationic part of compound **C3** (*m*/*z* 753 Da) with neutral losses explanation (B).

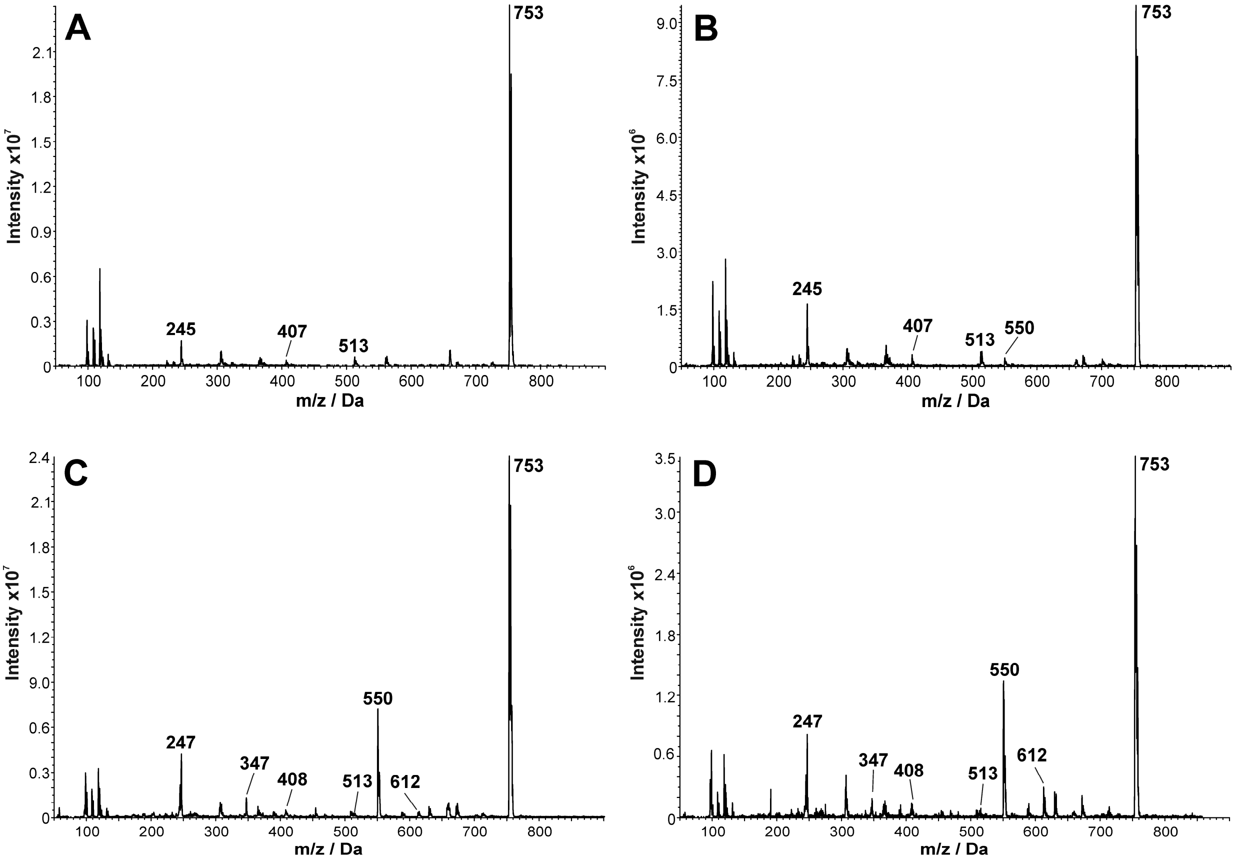

Figure S8. Positive-ion ESI mass spectra of compound **C3** dissolved in pure (A), 90% (B), 70% (C), and 50% (*v/v*) MeCN (D).

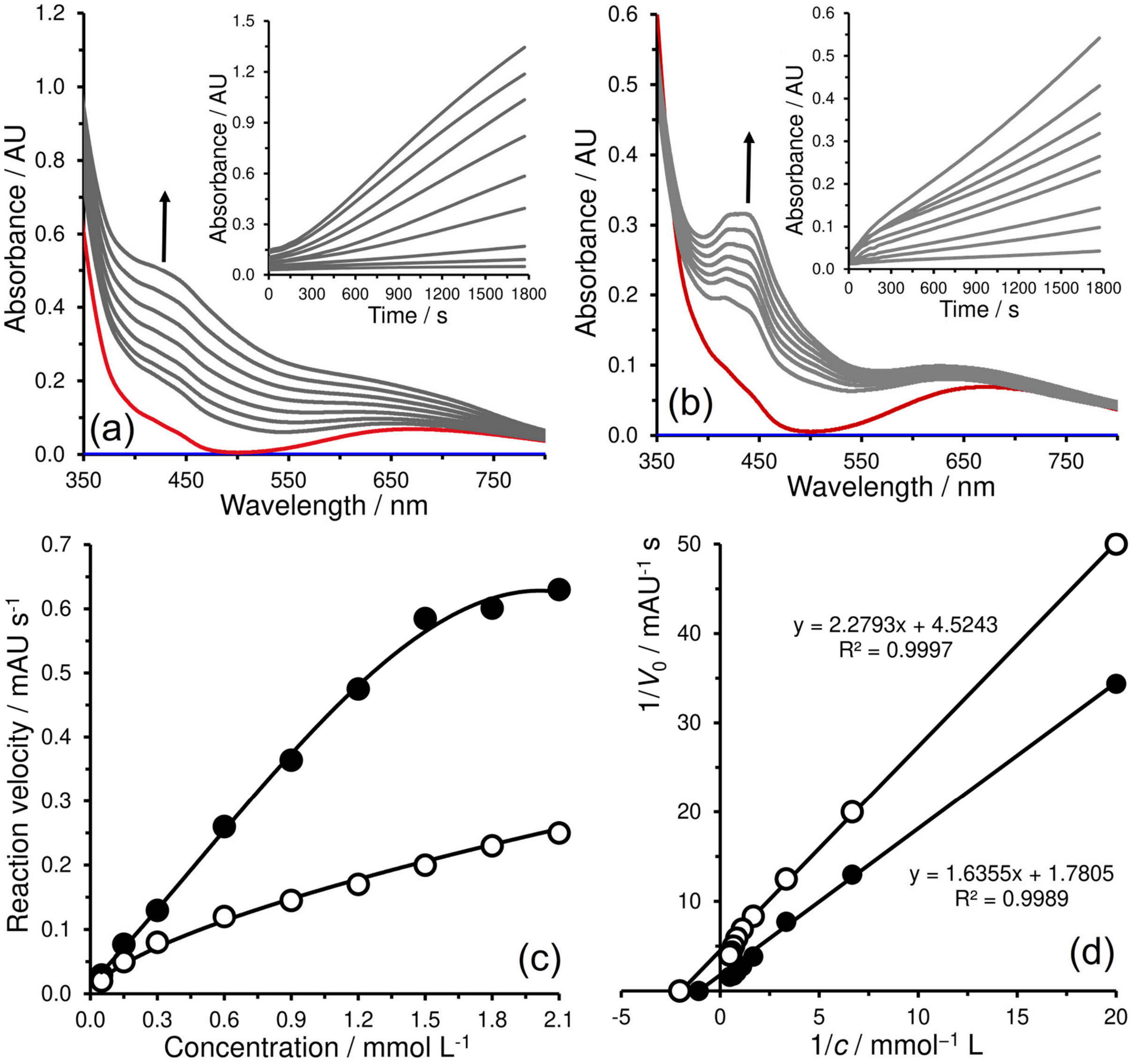

Figure S9. Spectral changes upon 500 μmol L⁻¹ 4-AP (a) or 4-A2CP (b) catalysed by 150 μmol L⁻¹ compound **C1** (red) in pure MeOH (blue spectrum). The inserted plots show kinetic curves obtained for the same concentration of **C1** catalysing the oxidation of 50, 150, 300, 600, 900, 1200, 1500, 1800, and 2100 μmol L⁻¹ 4-AP (a) or 4-A2CP (b) in MeOH at 430 nm. Typical Michaelis–Menten saturation curves for compound **C1** (c), showing the relation between the reaction rate and the concentration of 4-AP (curves with full) or 4-A2CP (curves with empty marks), and the appropriate Lineweaver–Burk plots (d).

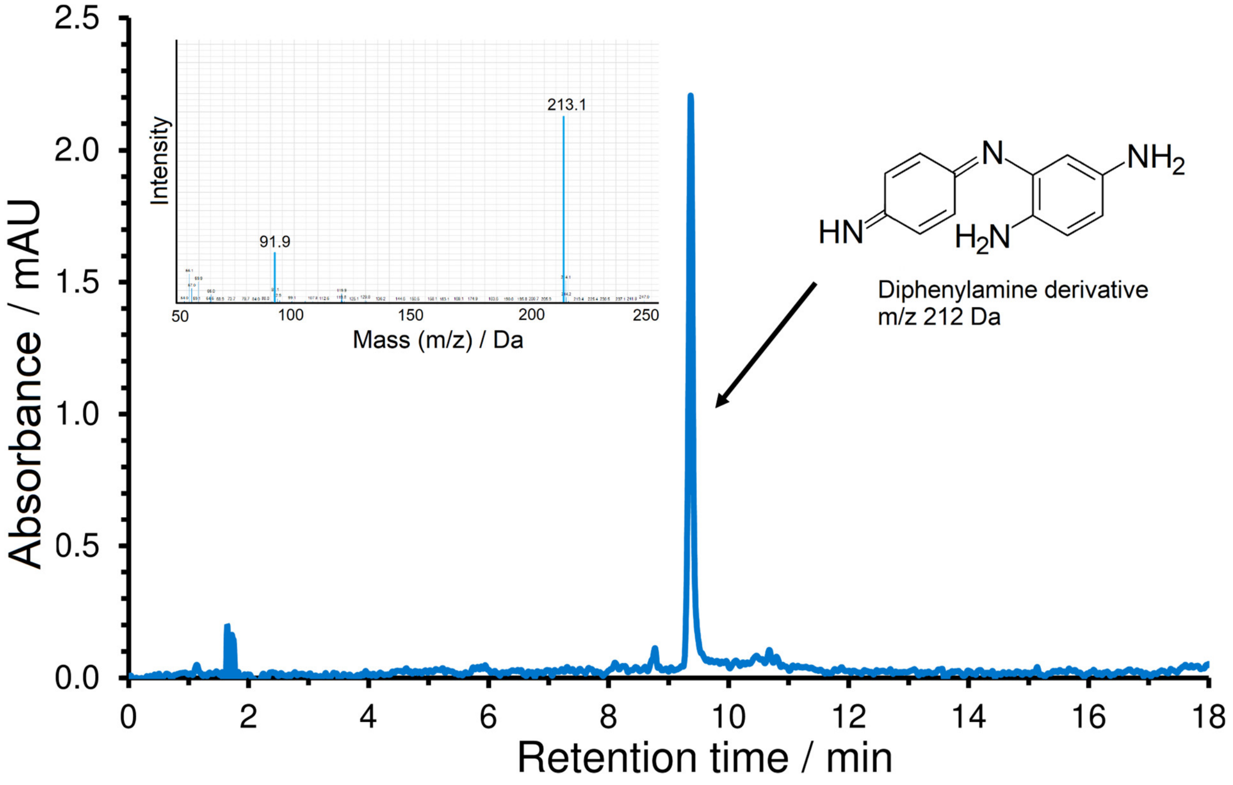

Figure S10. HPLC chromatogram of the reaction mixture obtained from the oxidation of PPD catalysed by compound **C1** after 12 hours. Experimental conditions: Kinetex C18 column (150 × 3 mm, 3 µm) tempered at 30 °C, gradient elution with gradient program. Mobile phase "A" contained 5 mmol L^{-1} ammonium acetate in deionized water and mobile phase "B" then pure methanol when both components were employed in the following sequences: 0 min - 5 % B; 15 min - 90 % B; 18 min - 90 % B. The flow rate of the mobile phase was set to 0.4 mL min^{-1} and the injection volume was 5 µL. Inserted figure shows the MS spectrum of the corresponding peak at retention time of 9.36 min (range 50-250 m/z; positive ion mode, fragmentation energy of 130 V).
